# Heteroleptic Coordination Environments in Metal-Mediated DNA G-Quadruplexes

**DOI:** 10.3389/fchem.2020.00026

**Published:** 2020-01-29

**Authors:** Philip M. Punt, Lukas M. Stratmann, Sinem Sevim, Lena Knauer, Carsten Strohmann, Guido H. Clever

**Affiliations:** Faculty of Chemistry and Chemical Biology, TU Dortmund University, Dortmund, Germany

**Keywords:** bioinorganic chemistry, coordination chemistry, DNA, G-quadruplex, DNAzymes

## Abstract

The presence of metal centers with often highly conserved coordination environments is crucial for roughly half of all proteins, having structural, regulatory, or enzymatic function. To understand and mimic the function of metallo-enzymes, bioinorganic chemists pursue the challenge of synthesizing model compounds with well-defined, often heteroleptic metal sites. Recently, we reported the design of tailored homoleptic coordination environments for various transition metal cations based on unimolecular DNA G-quadruplex structures, templating the regioselective positioning of imidazole ligandosides **L**^**I**^. Here, we expand this modular system to more complex, heteroleptic coordination environments by combining **L**^**I**^ with a new benzoate ligandoside **L**^**B**^ within the same oligonucleotide. The modifications still allow the correct folding of parallel tetramolecular and antiparallel unimolecular G-quadruplexes. Interestingly, the incorporation of **L**^**B**^ results in strong destabilization expressed in lower thermal denaturation temperatures *T*_*m*_. While no transition metal cations could be bound by G-quadruplexes containing only **L**^**B**^, heteroleptic derivatives containing both **L**^**I**^ and **L**^**B**^ were found to complex Cu^II^, Ni^II^, and Zn^II^. Especially in case of Cu^II^ we found strong stabilizations of up to Δ*T*_*m*_ = +34°C. The here shown system represents an important step toward the design of more complex coordination environments inside DNA scaffolds, promising to culminate in the preparation of functional metallo-DNAzymes.

## Introduction

Proteins are involved in a vast number of processes ranging from structural and regulatory functions to enzymatic reactions. Roughly half of all proteins depend on metal cations helping to maintain a desired folding or serving as catalytic centers or redox cofactors (Raven et al., 1999; Lu et al., 2009; Rubino and Franz, 2012). Which function the respective metal ion adopts is strongly dependent on its properties, including accessible spin states, oxidation potential, Lewis-acidity, and bioavailability (Holm et al., 1996; Waldron et al., 2009). These properties are further fine-tuned by a well-defined first and second coordination sphere. The former is directly involved in metal coordination and usually consists of mixtures of different donor functionalities. Typically involved in coordination are the amino acids histidine, glutamic/aspartic acid, methionine, cysteine, or the backbone amide groups (Holm et al., 1996; Degtyarenko, 2000; Shook and Borovik, 2010; Valdez et al., 2014). In contrast, the second coordination sphere is not directly involved in metal binding but regulates catalytic processes, proton or electron shuttling, substrate transport, and effects selectivity (Colquhoun et al., 1986; Degtyarenko, 2000; Waldron et al., 2009; Shook and Borovik, 2010; Zhao et al., 2013; Valdez et al., 2014; Cornish et al., 2016).

The design of artificial metallo-enzyme mimics with improved or novel properties is attracting increasing interest, but remains challenging. In the area of preparative bioinorganic chemistry, focus is set on small, multidentate chelate complexes, often requiring tedious multistep syntheses and only covering effects of the first coordination sphere (Samuel et al., 2010; Kanady et al., 2011; Anderson et al., 2013; Dicke et al., 2018). More biologically oriented approaches involve the replacement of natural metal cofactors with metal centers not known in nature. An example is the replacement of hemin in myoglobin with an iridium or rhodium porphyrin complex for enantioselective cyclopropanation reactions (Key et al., 2016; Litman et al., 2018). Another approach is embedding metal cofactors by covalent or non-covalent interactions into empty cavities of usually metal-free proteins. This was successfully applied in a series of examples enabling catalysis of the asymmetric transfer hydrogenation of imines (Wu et al., 2019), ring-closing metathesis (Jeschek et al., 2016), oxime (Drienovská et al., 2018), and hydrazine (Drienovská et al., 2018; Mayer et al., 2019) formation and hydration of alkenes (Drienovská et al., 2017). In contrast to the aforementioned examples, a more bottom up approach is the *de novo* design of new metallo-proteins by the precise arrangement of certain structural motifs to create a metal binding site (Raven et al., 1999; Lu et al., 2009; Rubino and Franz, 2012). In recent years, a more efficient alternative was developed based on small artificial peptoid structures. Due to their simple accessibility by solid phase synthesis and their capability to form well-ordered secondary structures, many examples were shown for selective metal binding and catalytic applications (Baskin and Maayan, 2016; Knight et al., 2017; Baskin et al., 2018; Ghosh et al., 2018).

Another type of biopolymers forming well-ordered secondary structures are oligonucleotides. In contrast to peptides, RNA and DNA only consist of four nucleotide building blocks, thus reducing the possibilities to create diverse coordination environments for a range of metal cations. To overcome this limitation, different strategies were developed to covalently or non-covalently anchor metal-chelating ligands inside DNA. Roelfes and co-workers pioneered the design of various oligonucleotides capable of Michael-Additions, Carbene transfer, *syn*-hydrations of alkenes or Diels-Alder reactions (Roelfes and Feringa, 2005; Coquière et al., 2007; Boersma et al., 2010a,b; Rioz-Martínez et al., 2016). Other groups used modified quadruplexes for sequence-specific DNA cleavage, light controlled thrombin catalysis or peroxidase mimicking DNAzymes (Xu et al., 2009; Ali et al., 2019; Wang et al., 2019). A difficulty of this approach lies in the largely unknown exact position and coordination environment of the catalytic centers. This difficulty could be overcome in the field of metal-mediated base pairing, where the hydrogen bonding interaction of canonical base pairs is replaced by metal coordination, leading to highly stabilized DNA structures (Mandal and Müller, 2017). While first examples included only the involvement of canonical bases (Katz, 1963), the field was later expanded by the incorporation of a variety of artificial nucleobases culminating in the development of programmable metal wires inside DNA duplexes (Tanaka et al., 2006; Clever et al., 2007; Mandal et al., 2016; Sandmann et al., 2019). Later, the concept was expanded from duplex to triplex DNA (Tanaka et al., 2002) and i-motifs (Abdelhamid et al., 2018), while we and others started to focus on G-quadruplexes (Miyoshi et al., 2007; Smith et al., 2012; Engelhard et al., 2013). The latter ones form from guanine-rich sequences where four G-residues cyclize to planar G-tetrads *via* Hoogsteen base pairing. Multiple G-tetrads form a G-quadruplex *via* π-π stacking interactions. Key to their high stability is the incorporation of a central cation—typically Na^+^ or K^+^ (Hänsel-Hertsch et al., 2017; Neidle, 2017). Our group was the first to report Cu^II^-mediated tetramolecular G-quadruplexes based on pyridine and imidazole ligands (Engelhard et al., 2013, 2018b; Punt and Clever, 2019a), aimed at a range of applications. For example, dinuclear systems were employed as Cu^II^-based EPR-rulers for accurate distance measurements (Engelhard et al., 2018a). We later expanded this concept to unimolecular G-quadruplexes, equipped with oligonucleotide loops which form cavities above the G-quadruplex stem in which the metal complexes are embedded (Engelhard et al., 2017). In a recent study, we further showed that these G-quadruplexes can act as robust templates to arrange different numbers of imidazole ligandosides, leading to fine-tuned affinities for a range of transition metal cations with respect to their preferred coordination environments (Punt and Clever, 2019b). While only homoleptic systems were investigated in that study, we herein expand the modular concept to heteroleptic systems with different donor functionalities. We introduce the design of mixed systems with imidazole and benzoate ligands, inspired by metallo-proteins, where the combination of imidazoles and carboxylate is often involved in metal coordination (e.g., in the 2-His-1-carboxylate facial triad) (Greenblatt et al., 1998; Koehntop et al., 2005). We show how this combination affects both, G-quadruplex stability and metal complexation.

## Results

**Figure 1:**
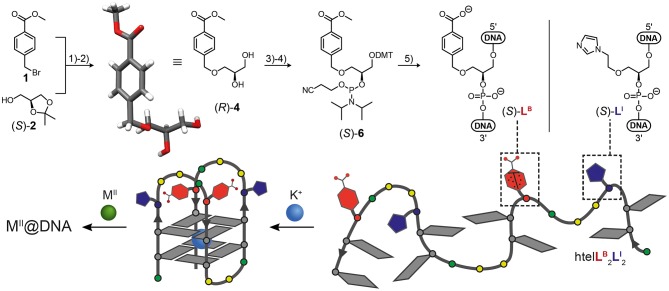
Synthesis of benzoate ligandoside **L**^B^ and molecular structure of ligandoside **L**^I^ (**top**). (1) NaH, CH_3_CN; (2) CH_3_COOH, THF/H_2_O; (3) DMT-Cl, DIPEA, DMAP, THF; (4) CEDIP-Cl, DIPEA, CH_2_Cl_2_; (5) automated solid-phase DNA synthesis. The single-crystal X-ray structure of the protected ligandoside (*R*)-**4** is shown. G-quadruplex formation (**bottom**) of htel**L**^B^_2_**L**^I^_2_ creates a heteroleptic coordination environment for transition metal ions (M = Co, Ni, Cu, Zn). Gray tiles: guanosine; red: ligandoside **L**^B^; blue: ligandoside **L**^I^; green circles: adenosine; yellow circles: thymidine. DMT = dimethoxytrityl; DIPEA = N,N-diisopropylethylamine; DMAP = N,N-dimethylaminopyridin; CEDIP-Cl = 2-cyanoethyl N,N-diisopropylchlorophosphoramidite.

In this study we report the incorporation of a new benzoate ligandoside **L**^B^ in combination with the known imidazole ligandoside **L**^I^. Both were incorporated in the (*S*) configuration as GNA (glycol nucleic acid) building blocks (Zhang et al., 2005, 2006) by solid phase synthesis into tetramolecular and unimolecular G-quadruplexes. The phosphoramidite of **L**^I^ was synthesized as previously described (Punt and Clever, 2019b). For the new benzoate ligand **L**^B^, a literature procedure was adopted (Engelhard et al., 2017). Accordingly, an initial nucleophilic attack of deprotonated solketal to methyl 4-(bromomethyl)benzoate followed by acidic deprotection reaction led to protected benzoate ligandoside (*R*)-**4**. Its structure and absolute configuration were confirmed by single-crystal X-ray diffraction (Figure 1). The primary hydroxyl group was DMT-protected (DMT = dimethoxytrityl) followed by a phosphorylation reaction yielding phosphoramidite building block (*S*)-**6**. DNA solid phase synthesis was then performed according to standard literature procedures with extended coupling times for the ligandosides **L**^I^ and **L**^B^ (see Supplementary Material for details). Coupling efficiencies for **L**^B^ and **L**^I^ were typically >99% per step. After solid phase synthesis, oligonucleotides were cleaved from the solid support and deprotected in 0.4 M NaOH in methanol/water (4:1) at 55°C for 16 h. Standard deprotection with concentrated ammonium hydroxide was avoided due to the risk of forming amides instead of carboxylates from the benzoate esters. After reversed-phase HPLC purification, oligonucleotides were desalted and DMT-groups removed using C18 *SepPak* cartridges and aq. TFA (2%). The oligonucleotides were then lyophilized at stored at −20°C.

**Figure 2:**
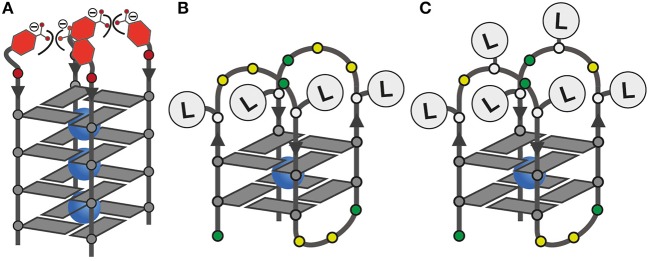
**(A)** Schematic representation of the tetramolecular G-quadruplex (**L**^B^G_4_)_4_ with the proposed repulsive effect of negatively charged **L**^B^; **(B,C)** ligand positions in unimolecular G-quadruplexes with four or six incorporated ligands.

Since **L**^I^ had already been established in tetramolecular and unimolecular G-quadruplexes, we first investigated the influence of **L**^B^ in the tetramolecular G-quadruplex (**L**^B^G_4_)_4_. Clear formation of a parallel G-quadruplex was observed by CD spectroscopy with a positive Cotton effect around ~260 nm (see Figure S25). Thermal denaturation experiments showed a melting temperature *T*_*m*_ of 27°C which was significantly lower compared to previously reported (**L**^I^G_4_)_4_ (*T*_*m*_ = 36°C; Punt and Clever, 2019b). Since **L**^B^ and **L**^I^ are sharing the same backbone modification, we ascribe this destabilization to a repulsive effect between the negatively charged benzoates and phosphates (Figure 2). Next, the interaction of (**L**^B^G_4_)_4_ with a series of transition metal cations was investigated. In contrast to (**L**^I^G_4_)_4_ which was shown to complex Cu^II^, Ni^II^, Co^II^, and Zn^II^, no signs for metal complexation in (**L**^B^G_4_)_4_ were observed (see Figures S3, S4). This may be explained by the harder character of the benzoate ligand, competing with hard ligands such as the contained chloride, cacodylate buffer or phosphate backbones. However, even for hard and oxophilic transition metal cations, including Gd^III^ and Ce^III^, no interactions were found.

**Figure 3:**
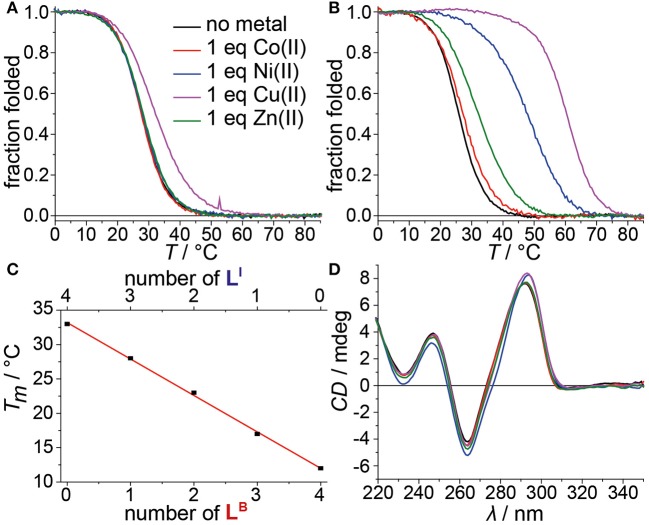
Melting curves of **(A)** htel**L**^B^**L**^I^_3_ and **(B)** htel**L**^B^_2_**L**^I^_4_ in absence or presence of different transition metal cations. **(C)** Linear dependence of thermal stabilities of htel**L**^I^_4_, htel**L**^B^**L**^I^_3_, htel**L**^B^_2_**L**^I^_2_, htel**L**^B^_3_**L**^I^, and htel**L**^B^_4_ depending on the number of incorporated **L**^B^. **(D)** CD spectra of htel**L**^B^_2_**L**^I^_4_ in absence or presence of different transition metal cations.

Mixing ligands in tetramolecular G-quadruplexes leads to statistical mixtures, which makes it challenging to design distinct heteroleptic coordination environments (see Supplementary Material for details). On the other hand, the folding of unimolecular G-quadruplexes into discrete topologies enables programmable ligand arrangements. Consequently, we moved forward to incorporate **L**^B^ in unimolecular G-quadruplexes. At first, **L**^B^ was incorporated four times in htel**L**^B^_4_. Similar to (**L**^B^G_4_)_4_, incorporation of **L**^B^ caused strong destabilization (*T*_*m*_ = 12°C) compared to htel**L**^I^_4_ (*T*_*m*_ = 33°C). Successive replacement of **L**^B^ with **L**^I^ was accompanied with a linear increase in stabilization for each replacement (htel**L**^B^_3_**L**^I^ *T*_*m*_ = 17°C, htel**L**^B^_2_**L**^I^_2_ *T*_*m*_ = 23°C, htel**L**^B^**L**^I^_3_ *T*_*m*_ = 28°C), highlighting the additive destabilizing effect of **L**^B^ (Figure 3). CD spectroscopy of htel**L**^B^_4_, htel**L**^B^_3_**L**^I^, htel**L**^B^_2_**L**^I^_2_, and htel**L**^B^**L**^I^_3_ showed clear signatures corresponding to an antiparallel G-quadruplex topology with a positive Cotton effect around ~294 nm in all cases (see Figures S26, S27). This is consistent with the previous observations for homoleptic G-quadruplexes containing only **L**^I^. Next, the interaction with different transition metal cations was investigated. As for (**L**^B^G_4_)_4_, for htel**L**^B^_4_, htel**L**^B^_3_**L**^I^, and htel**L**^B^_2_**L**^I^_2_, thermal denaturation experiments showed no signs for interaction with the examined transition metal cations (Cu^II^, Ni^II^, Zn^II^, Co^II^, V^IV^O). Pleasingly, this changed for htel**L**^B^**L**^I^_3_ that showed a weak but distinct stabilization after addition of 1 equiv. of Cu^II^ (Δ*T*_*m*_ = + 4°C). Additional equivalents resulted in no further stabilization consistent with a specific binding of Cu^II^. CD spectroscopy further confirmed retention of a clear antiparallel topology (see Figures S6, S7, S11–S16).

After we could show that at least three imidazole ligands are required to complex Cu^II^, we moved forward to a new series of sequences with six incorporated ligands (htel**L**^B^_4_**L**^I^_2_, htel**L**^B^_3_**L**^I^_3_, htel**L**^B^_2_**L**^I^_4_). Again, the formation of G-quadruplexes with a clear antiparallel topology was observed by CD spectroscopy (see Figures S28, S29). Likewise, comparison of the thermal stabilities showed the destabilizing effect of **L**^B^ (htel**L**^B^_4_**L**^I^_2_ *T*_*m*_ = 17°C, htel**L**^B^_3_**L**^I^_3_ *T*_*m*_ = 26°C, htel**L**^B^_2_**L**^I^_4_ *T*_*m*_ = 26°C), however, not in the linear fashion as observed for the series htel**L**^B^_4−n_**L**^I^_n_ (*n* = 0–4). For the examined set of six-ligand-containing sequences, however, direct *T*_*m*_ comparison is not appropriate due to the chosen modification pattern (see Table 1). When investigating the interaction with metal cations, for htel**L**^B^_4_**L**^I^_2_, a clear stabilization after addition of Cu^II^ (Δ*T*_*m*_ = + 6°C) was observed. Considering that for htel**L**^B^_2_**L**^I^_2_ almost no stabilization was observed (Δ*T*_*m*_ = + 1°C), we conclude that in htel**L**^B^_4_**L**^I^_2_ an involvement of one or two ligandosides **L**^B^ into metal coordination is very likely. When further replacing **L**^B^ with **L**^I^ as in htel**L**^B^_3_**L**^I^_3_ and htel**L**^B^_2_**L**^I^_4_, the Cu^II^-mediated thermal stabilization successively increased from Δ*T*_*m*_ = + 9°C (htel**L**^B^_3_**L**^I^_3_) to Δ*T*_*m*_ = + 34°C (htel**L**^B^_2_**L**^I^_4_). This extremely high thermal stabilization is unprecedented for unimolecular G-quadruplexes and much higher compared to the reported G-quadruplexes htel**L**^I^_6_ (Δ*T*_*m*_ = + 18°C) and htel**L**^I^_4_A (Δ*T*_*m*_ = + 23°C) (Punt and Clever, 2019b).

**Table 1:** Sequences investigated in this study and respective denaturation temperatures *T*_*m*_ (and Δ*T*_*m*_) in absence and presence of 1 equiv. of Cu^II^, Ni^II^, Zn^II^, Co^II^ (assumed to be oxidized to Co^III^ under the experimental conditions).

**Name**	**Sequence 5^**′**^ 3^**′**^**	**No metal**	**Co^**II**^**	**Ni^**II**^**	**Cu^**II**^**	**Zn^**II**^**
**L**^I^${\text{G}}_{\text{n}}^{\left[\text{a}\right]}$	**L**^I^**G**_n_	36	63 (+27)	73 (+37)	76 (+40)	52 (+16)
**L**^B^G_n_	**L**^B^**G**_n_	27	27 (0)	27 (0)	27 (0)	27 (0)
htel**L**^I^_4_A^[a]^	AGG **L**^I^TT A**L**^I^G GTT AGG **L**^I^TT A**L**^I^G G	33	35 (+2)	45 (+12)	56 (+23)	36 (+3)
htel**L**^B^_4_	AGG **L**^B^TT A**L**^B^G GTT AGG **L**^B^TT A**L**^B^G G	12	12 (0)	12 (0)	12 (0)	12 (0)
htel**L**^I^_4_B	AGG **L**^I^TT T**L**^I^G GTT AGG **L**^I^TT T**L**^I^G G	40	40 (0)	46 (+6)	60 (+20)	40 (0)
htel**L**^B^_3_**L**^I^	AGG **L**^I^TT A**L**^B^G GTT AGG **L**^B^TT A**L**^B^G G	17	17 (0)	17 (0)	17 (0)	17 (0)
htel**L**^B^_2_**L**^I^_2_	AGG **L**^I^TT A**L**^B^G GTT AGG **L**^I^TT A**L**^B^G G	23	23 (0)	23 (+0)	24 (+1)	23 (0)
htel**L**^B^**L**^I^_3_	AGG **L**^I^TT A**L**^I^G GTT AGG **L**^I^TT A**L**^B^G G	28	28 (0)	28 (+0)	32 (+4)	28 (0)
htel${\text{L}}_{6}^{\text{I}[\text{a}]}$	AGG **L**^I^T**L**^I^ T**L**^I^G GTT AGG **L**^I^T**L**^I^ T**L**^I^G G	36	44 (+8)	59 (+23)	54 (+18)	44 (+8)
htel**L**^B^_4_**L**^I^_2_	AGG **L**^B^T**L**^I^ T**L**^B^G GTT AGG **L**^B^T**L**^I^ T**L**^B^G G	17	17 (0)	18 (+1)	23 (+6)	18 (+1)
htel**L**^B^_3_**L**^I^_3_	AGG **L**^B^T**L**^I^ T**L**^I^G GTT AGG **L**^B^T**L**^B^ T**L**^I^G G	26	25 (−1)	26 (+0)	35 (+9)	31 (+5)
htel**L**^B^_2_**L**^I^_4_	AGG **L**^I^T**L**^B^ T**L**^I^G GTT AGG **L**^I^T**L**^B^ T**L**^I^G G	26	27 (+1)	48 (+22)	60 (+34)	32 (+6)

*Marked in b font are the incorporated ligandosides **L**^B^ and **L**^I^. Conditions: 4 μM (tetramolecular) or 1.88 μM (unimolecular) ssDNA, 100 mM NaCl (tetramolecular) or KCl (unimolecular), 10 mM lithium cacodylate buffer (LiCaCo) pH 7.2 and, if present, 1 equiv. transition metal cation (with respect to the folded G-quadruplex). [a] Punt and Clever (2019b)*.

**Figure 4:**
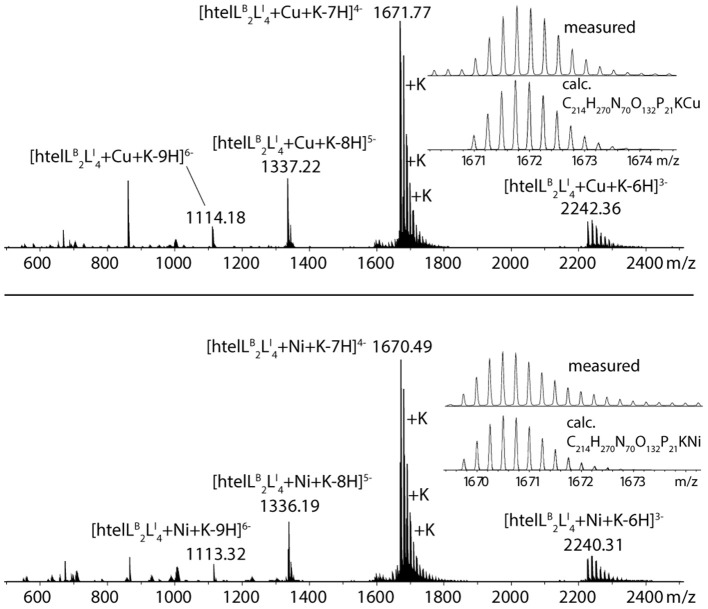
Native ESI-MS spectra of htel**L**^B^_2_**L**^I^_4_ in complex with Cu^II^ (**top**) and Ni^II^ (**bottom**).

The formation of 1:1 complexes for htel**L**^B^_2_**L**^I^_4_ with Cu^II^ and Ni^II^ was further confirmed by native ESI mass spectrometry. To understand whether a G-quadruplex is folded or unfolded in the gas phase, the intrinsic property of G-quadruplexes is exploited that in their folded state they always bind *n*−1 potassium ions (where *n* = number of G-tetrads). For a folded G-quadruplex with two G-tetrads, a main signal corresponding to the adduct with one distinct potassium ion would be expected, followed by a statistical distribution of adducts with further unspecifically bound potassium cations. On the other hand, for an unfolded G-quadruplex, the main signal would correspond to the mass of the DNA strand without potassium ions. The mass spectrum shows a main signal corresponding to [htel**L**^B^_2_**L**^I^_4_+Cu+K-7H]^4−^ (Figure 4), thus strongly indicating a folded G-quadruplex coordinating to a Cu^II^ or Ni^II^ ion in the gas phase (D'Atri et al., 2015; Lecours et al., 2017).

Jahn-Teller-distorted Cu^II^ usually favors the coordination of four strongly associated ligands in a square planar geometry, with two additional ligands more loosely bound in axial positions (Halcrow, 2012). After proving a 1:1 complex for htel**L**^B^_2_**L**^I^_4_ and Cu^II^, the question was if all six ligands are participating in metal coordination or if only **L**^I^ is involved. Therefore, a new sequence htel**L**^I^_4_B was synthesized where **L**^B^ was replaced with thymidines. Addition of Cu^II^ led to a thermal stabilization of Δ*T*_*m*_ = + 20°C, much lower compared to htel**L**^B^_2_**L**^I^_4_ (Δ*T*_*m*_ = + 34°C). However, when looking at the absolute melting temperature *T*_*m*_ in presence of Cu^II^, one notices that they are the same for both sequences (htel**L**^B^_2_**L**^I^_4_ *T*_*m*_ = 60°C, htel**L**^I^_4_B *T*_*m*_ = 60°C). This could mean that Cu^II^ coordination by htel**L**^B^_2_**L**^I^_4_ simply compensates the destabilizing effect of **L**^B^ and no benzoate ligand was involved in Cu^II^ coordination. Further studies are required to shed light on this question.

Besides Cu^II^, the addition of Zn^II^ and Ni^II^ to htel**L**^B^_2_**L**^I^_4_ and htel**L**^B^_3_**L**^I^_3_ led to thermal stabilizations. These results were highly intriguing for two reasons. Quadruplex htel**L**^B^_2_**L**^I^_4_ was significantly more stabilized with Ni^II^ (Δ*T*_*m*_ = + 22°C) compared to Zn^II^ (Δ*T*_*m*_ = + 6°C). However, in htel**L**^B^_3_**L**^I^_3_, the opposite effect was observed, showing a higher stabilization after Zn^II^ addition (Δ*T*_*m*_ = + 5°C), while for Ni^II^ no complexation was observed. This adds to the established variation of ligand number and position a third layer to our system to fine-tune metal affinities by the introduction of heteroleptic systems. As last question, we were interested whether Zn^II^ in htel**L**^B^_3_**L**^I^_3_ is coordinated by one or more benzoates. Interestingly, other sequences shown to complex Zn^II^ (htel**L**^I^_4_A Δ*T*_*m*_ = + 3°C, htel**L**^I^_6_ Δ*T*_*m*_ = + 8°C) always contain at least four counts of **L**^I^. Since in htel**L**^B^_3_**L**^I^_3_ only three **L**^I^ were available, we conclude that an involvement of **L**^B^ in coordination to the Zn^II^ cation is likely.

## Conclusion

A new benzoate-based ligandoside **L**^B^ was established in tetramolecular and unimolecular G-quadruplex structures. Homoleptic G-quadruplex (**L**^B^G_4_)_4_ was found to form a clear parallel topology. Its thermal stability indicated a strongly destabilizing effect of **L**^B^ compared to **L**^I^ which was attributed to an accumulation of negative charges. Also, no interactions between a series of transition metal cations and (**L**^B^G_4_)_4_ were found. Similarly, for the unimolecular G-quadruplex htel**L**^B^_4_, a destabilizing effect of **L**^B^ and no interactions with transition metal cations were observed. The successive replacement of **L**^B^ with **L**^I^ in htel**L**^B^_3_**L**^I^, htel**L**^B^_2_**L**^I^_2_, htel**L**^B^**L**^I^_3_, and htel**L**^I^_4_ resulted in a linear increase of the thermal stability. In addition, for htel**L**^B^**L**^I^_3_, a weak thermal stabilization after addition of 1 equiv. Cu^II^ indicated specific binding.

When moving to systems with six incorporated ligands, a tremendously high thermal stabilization was observed after addition of Cu^II^ to htel**L**^B^_2_**L**^I^_4_ (Δ*T*_*m*_ = + 34°C). In comparison, for htel**L**^I^_4_B, addition of Cu^II^ resulted in a stabilization of only Δ*T*_*m*_ = + 20°C. However, the absolute melting temperatures *T*_*m*_ of htel**L**^B^_2_**L**^I^_4_ (*T*_*m*_ = 60°C) and htel**L**^I^_4_B (*T*_*m*_ = 60°C) are the same, indicating that Cu^II^ complexation is rather compensating the destabilizing effect of **L**^B^. More interesting were the results for htel**L**^B^_2_**L**^I^_4_ and htel**L**^B^_3_**L**^I^_3_ after addition of Zn^II^ and Ni^II^, respectively. Htel**L**^B^_2_**L**^I^_4_ was significantly more stabilized by Ni^II^ (Δ*T*_*m*_ = + 22°C) compared to Zn^II^ (Δ*T*_*m*_ = + 6°C). However, in htel**L**^B^_3_**L**^I^_3_, the opposite effect was observed, showing a higher stabilization after Zn^II^ addition (Δ*T*_*m*_ = + 5°C) while for Ni^II^ no complexation was found. This expands our toolbox to design tailored binding sites for various transition metal cations. Previously, we had shown to fine-tune coordination environments by varying position and number of ligands. Here, we expand this approach by combining two ligandosides, **L**^B^ and **L**^I^, which we regard as an important step for the design of metal-selective G-quadruplexes with application in diagnostics, selective catalysis, and DNA nanotechnology.

## Data Availability Statement

The datasets generated for this study can be found in the Cambridge Crystallographic Data Center under the CCDC identifier 1961648.

## Author Contributions

PP and LS conducted all syntheses and DNA experiments. SS contributed to the tetramolecular systems. LK and CS contributed the X-ray structure of compound 4. PP, LS, and GC designed the study, conceived the experiments, analyzed the data, and authored the manuscript.

### Conflict of Interest

The authors declare that the research was conducted in the absence of any commercial or financial relationships that could be construed as a potential conflict of interest.
