# Supplementary material for: Heteroleptic Coordination Environments in Metal-Mediated DNA G-Quadruplexes
Source: Front Chem. 2020 Jan 29;8:26. doi: 10.3389/fchem.2020.00026 (PMC7000376; doi:10.3389/fchem.2020.00026)
Supplement: Supplementary file 1 [file Data_Sheet_1.PDF]

Table of Contents

1 Synthesis 2

1.1 Synthesis of methyl (*S*)-4-(((2,2-dimethyl-1,3-dioxolan-4-yl)methoxy)methyl)benzoate (*S*)-3 3

1.2 Synthesis of methyl (*R*)-4-((2,3-dihydroxypropoxy)methyl)benzoate (*R*)-4 4

1.3 Synthesis of methyl (*S*)-4-((3-(bis(4-methoxyphenyl)(phenyl)methoxy)-2-hydroxypropoxy)methyl)benzoate (*S*)-5 5

1.4 Synthesis of methyl 4-(((2*S*)-3-(bis(4-methoxyphenyl)(phenyl)methoxy)-2-(((2-cyanoethoxy)(diisopropylamino)phosphaneyl)oxy)propoxy)methyl)benzoate (*S*)-6 6

1.5 Synthesis of (*R*)-4-((2,3-dihydroxypropoxy)methyl)benzoic acid (*R*)-7 6

2 Single-Crystal X-ray Diffractional Analysis of (*R*)-4 7

3 Oligonucleotide Synthesis and Purification 9

4 Analytics of Oligonucleotides 11

4.1 Analytical RP-HPLC 11

4.1 Mass Spectrometry 13

5 UV-based Thermal Denaturation Studies 17

5.1 Sample Preparation 17

5.2 Spectrometer and Methods 17

5.3 Thermal Denaturation Profiles and Thermal Difference Spectra 18

6 CD Spectroscopy 30

6.1 Sample Preparation 30

6.2 Spectrometer and Methods 30

6.3 CD Spectra 30

7 Native ESI Mass Spectrometry 33

7.1 Sample Preparation 33

7.2 Spectrometer and Methods 33

7.3 Native ESI Mass Spectra 33

8 Mixtures of Ligands in Tetramolecular G-Quadruplexes 35

9 References 37

1 Synthesis

Chemicals and solvents were purchased from *Sigma Aldrich*, *Acros Organics*, *Carl Roth*, *TCI Europe*, *ABCR* or other suppliers and used as received. If necessary, reactions were carried out under an inert nitrogen atmosphere using standard Schlenk techniques or in a *GS-systems* Glovebox. Dry solvents were purified and dried over absorbent-filled columns on a *GS-Systems* solvent purification system.
Reactions were monitored with thin layer chromatography (TLC), using silica coated aluminium plates (*Merck*, silica 60, fluorescence indicator F254, thickness 0.25 mm). For the column chromatography, silica (*Merck*, silica 60, 0.02–0.063 mesh ASTM) was used as the stationary phase.
NMR spectra were recorded on a *Bruker AV 400 Avance III HD NanoBay*, *AV 600 Avance III HD* or *AV 700 Avance III HD* spectrometer. Chemical shifts δ are given in ppm. For ^{1}H and ^{13}C NMR spectra, chemical shifts were calibrated to the solvent lock signal. ^{31}P NMR chemical shifts are given relative to 85% H_3PO_4 (external reference). Signal multiplicities are composed of the following abbreviations: s (singlet), d (doublet), t (triplet), q (quartet), m (multiplett).
Mass spectrometry was performed on a *Bruker ESI-timsTOF* mass spectrometer. For calibration of the TOF device, *Agilent ESI-Low Concentration Tuning Mix* was used. Melting points were determined on a *Stuart SMP30* melting point apparatus. Elemental analyses were performed on an *Elementar vario MICRO cube*. IR spectra were recorded on a *PerkinElmer Spectrum Two* FT-IR spectrometer with $LiTaO_3$ MIR detector and an optical system with KBr windows. UV spectra were recorded on a *Jasco V-750* spectrometer.
The phosphoramidite for ligand $\mathbf{L^I}$ was synthesized as previously reported (Punt and Clever, 2019). The artificial nucleoside (*R*)-**7** and the respective phosphoramidite (*S*)-**6** needed for the incorporation of ligand $\mathbf{L^B}$ into oligonucleotides were synthesized following the route in scheme S1.

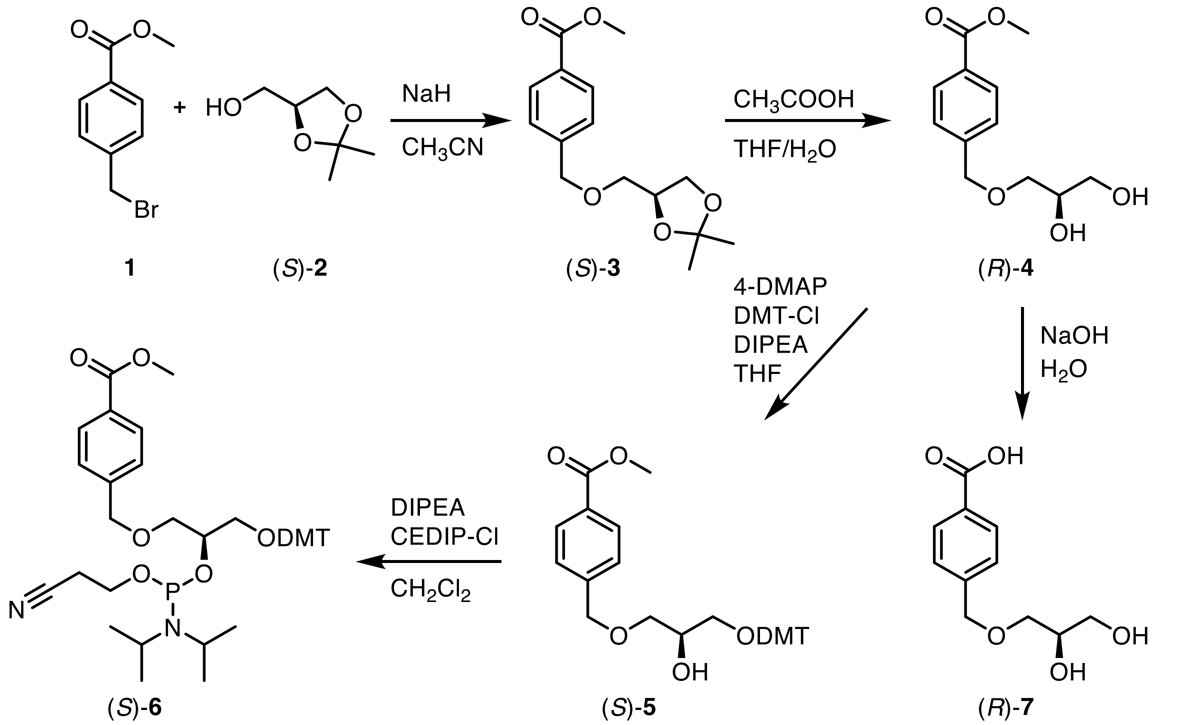

Scheme S1: Synthesis of the artificial nucleoside (*R*)-**7** and phosphoramidite (*S*)-**6** for ligand **L^{B}**.

1.1 Synthesis of methyl (*S*)-4-(((2,2-dimethyl-1,3-dioxolan-4-yl)methoxy)-methyl)benzoate (*S*)-3

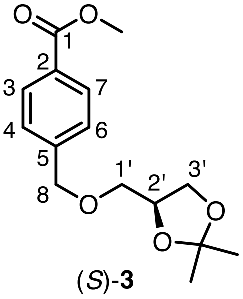

(*S*)-**3**

To a suspension of sodium hydride (60% in mineral oil; 0.36 g, 8.42 mmol, 1.1 equiv.) in dry acetonitrile (35 mL) was added (*S*)-(2,2-dimethyl-1,3-dioxolan-4-yl)methanol ((*S*)-**2**, 1.00 mL, 8.02 mmol, 1.0 equiv.) dropwise. After 30 min of stirring, the mixture was cooled to 0 °C and methyl 4-(bromomethyl)-benzoate (**1**, 1.84 g, 8.02 mmol, 1.0 equiv.) was added. The reaction mixture was allowed to warm up to room temperature and stirred overnight. The solvent was removed under reduced pressure and the obtained solid was taken up in ethyl acetate (50 mL). After filtration, the filtrate was washed with water (50 mL), the aqueous layer was extracted with ethyl acetate (3x 30 mL) and the combined organic layers were dried over magnesium sulfate. The solvent was removed under reduced pressure to afford an oil which was purified by column chromatography (*n*-pentane/ethyl acetate, 5:1) to obtain the product (*S*)-**3** as a colorless oil (1.38 g, 4.93 mmol, 61%).

^{1}H NMR (700 MHz, chloroform-*d*): δ = 7.99 (d, J = 8.4 Hz, 2H, 3-H and 7-H), 7.38 (d, J = 8.5 Hz, 2H, 4-H and 6-H), 4.62 (d, J = 12.9 Hz, 1H, 8-H_a), 4.59 (d, J = 12.8 Hz, 1H, 8-H_b), 4.32 – 4.27 (m, 1H, 2'-H), 4.05 (dd, J = 8.3 Hz, J = 6.5 Hz, 1H, 3'-H_a), 3.89 (s, 3H, $CO_2C\underline{H}_3$), 3.73 (dd, J = 8.3 Hz, J = 6.3 Hz, 1H, 3'-H_b), 3.55 (dd, J = 9.9 Hz, J = 5.7 Hz, 1H, 1'-H_a), 3.49 (dd, J = 9.9 Hz, J = 5.3 Hz, 1H, 1'-H_b), 1.40 (s, 3H, $C(C\underline{H}_3)_2$), 1.35 (s, 3H, $C(C\underline{H}_3)_2$).

^{13}C{^{1}H} NMR (176 MHz, chloroform-*d*): δ = 166.9 (C1), 143.4 (C5), 129.8 (C3 and C7), 129.5 (C2), 127.3 (C4 and C6), 109.6 ($\underline{C}(CH_3)_2$), 74.8 (C2'), 72.9 (C8), 71.5 (C1'), 66.8 (C3'), 52.1 ($CO_2\underline{C}H_3$), 26.8 ($C(\underline{C}H_3)_2$), 25.5 ($C(\underline{C}H_3)_2$).

HR-ESI MS (positive mode, MeCN): *m/z* calc. for $C_{15}H_{20}O_5Na$ 303.1203 $[M+Na]^+$, found 303.1198.

Elemental analysis (%): Calc. for $C_{15}H_{20}O_5$: C 64.3, H 7.2; found: C 64.0, H 7.3.

1.2 Synthesis of methyl (*R*)-4-((2,3-dihydroxypropoxy)methyl)benzoate (*R*)-4

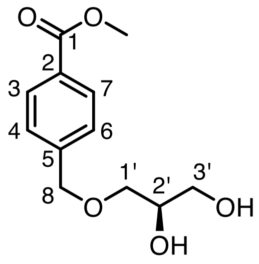

(*R*)-**4**

A solution of compound (*S*)-**3** (1.05 g, 3.75 mmol, 1.0 equiv.) in of tetrahydrofuran (26 mL) and 50% aqueous acetic acid (26 mL) was stirred overnight at 60 °C. The solvents were removed under reduced pressure to afford a solid which was purified by column chromatography (dichloromethane/methanol, 10:1) to afford the product (*R*)-**4** as a colorless crystalline material (0.86 g, 3.60 mmol, 96%) with a melting point at 84 °C.

^{1}H NMR (400 MHz, chloroform-*d*): δ = 8.02 (d, J = 8.3 Hz, 2H, 3-H and 7-H), 7.38 (d, J = 8.2 Hz, 2H, 4-H and 6-H), 4.60 (s, 2H, 8-H), 3.96 – 3.88 (m, 4H, 2'-H and $CO_2C\underline{H}_3$), 3.72 (dd, J = 11.4 Hz, J = 3.9 Hz, 1H, 3'-H_a), 3.64 (dd, J = 11.4 Hz, J = 5.6 Hz, 1H, 3'-H_b), 3.61 – 3.53 (m, 2H, 1'-H_a and 1'-H_b), 2.46 (br s, 2H, 2'-O$\underline{H}$ and 3'-O$\underline{H}$).

^{13}C{^{1}H} NMR (101 MHz, chloroform-*d*): δ = 167.0 (C1), 143.1 (C5), 129.9 (C3 and C7), 129.7 (C2), 127.4 (C4 and C6), 73.1 (C8), 72.2 (C1'), 70.8 (C2'), 64.1 (C3'), 52.3 ($CO_2\underline{C}H_3$).

HR-ESI MS (positive mode, MeCN): *m/z* calc. for $C_{12}H_{16}O_5Na$ 263.0890 $[M+Na]^+$, found 263.0885.

Elemental analysis (%): Calc. for $C_{12}H_{16}O_5$: C 60.0, H 6.7; found: C 59.9, H 6.7.

IR (ATR): $\tilde{\nu}$ = 3264, 2942, 2864, 1719, 1431, 1415, 1389, 1276, 1207, 1194, 1179, 1103, 1070, 1048, 1025, 1014, 954, 933, 866, 851, 763,712,529 cm^{-1}.

1.3 Synthesis of methyl (*S*)-4-((3-(bis(4-methoxyphenyl)(phenyl)methoxy)-2-hydroxypropoxy)methyl)benzoate (*S*)-5

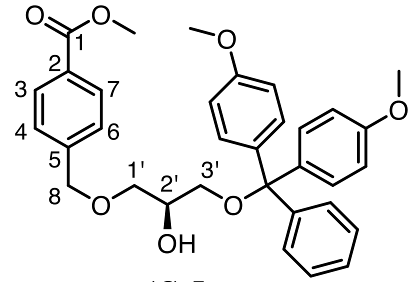

(*S*)-**5**

A solution of compound (*R*)-**4** (1.18 g, 4.93 mmol, 1.0 equiv.) and 4-dimethylaminopyridine (0.04 g, 0.30 mmol, 0.06 equiv.) in dry tetrahydrofuran (20 mL) was cooled to 0 °C and a solution of 4,4'-dimethoxytrityl chloride (2.01 g, 5.92 mmol, 1.2 equiv.) in dry tetrahydrofuran (10 mL) was added. After addition of *N*,*N*-diisopropylethylamine (1.68 mL, 9.86 mmol, 2.0 equiv.), the reaction mixture was allowed to warm up to room temperature and stirred overnight. The formed solid was filtered off and washed with ethyl acetate (15 mL). The solvents of the filtrate were removed under reduced pressure and the obtained yellow oil was purified by column chromatography (*n*-pentane/ethyl acetate, 5:1 + 0.5% triethylamine to 2:1 + 0.5% triethylamine) to give product (*S*)-**5** as a yellow oil (1.95 g, 3.59 mmol, 73%).

^{1}H NMR (600 MHz, methylene chloride-*d*$_2$): δ = 8.00 – 7.96 (m, 2H, 3-H and 7-H), 7.44 – 7.41 (m, 2H, DMT-$\underline{H}_{ar}$), 7.38 – 7.34 (m, 2H, 4-H and 6-H), 7.32 – 7.26 (m, 6H, DMT-$\underline{H}_{ar}$), 7.24 – 7.20 (m, 1H, DMT-$\underline{H}_{ar}$), 6.85 – 6.80 (m, 4H, DMT-$\underline{H}_{ar}$), 4.57 (s, 2H, 8-H), 3.99 – 3.93 (m, 1H, 2'-H), 3.89 (s, 3H, $CO_2C\underline{H}_3$), 3.77 (s, 6H, DMT-$OC\underline{H}_3$), 3.60 (dd, *J* = 9.6 Hz, 4.4, 1H, 1'-H_a), 3.57 (dd, *J* = 9.6 Hz, 6.0, 1H, 1'-H_b), 3.18 (d, *J* = 5.4 Hz, 2H, 3'-H), 2.36 (d, *J* = 5.1 Hz, 1H, 2'-O$\underline{H}$).

^{13}C{^{1}H} NMR (151 MHz, methylene chloride-*d*$_2$): δ = 167.2 (C1), 159.2 (DMT-C_{ar}), 145.6 (DMT-C_{ar}), 144.2 (C5), 136.5 (DMT-C_{ar}), 130.6 (DMT-C_{ar}), 130.0 (C3 and C7), 130.0 (C2), 128.6 (DMT-C_{ar}), 128.3 (DMT-C_{ar}), 127.7 (C4 and C6), 127.3 (DMT-C_{ar}), 113.6 (DMT-C_{ar}), 86.6 (DMT-$\underline{C}(Ar)_3$), 73.1 (C8), 72.5 (C1'), 70.5 (C2'), 65.0 (C3'), 55.7 (DMT-$O\underline{C}H_3$), 52.5 ($CO_2\underline{C}H_3$).

HR-ESI MS (positive mode, MeCN): *m/z* calc. for $C_{21}H_{19}O_2$ 303.1380 [DMT]$^+$, $C_{33}H_{34}O_7Na$ 565.2197 [M+Na]$^+$, $C_{33}H_{34}O_7K$ 581.1936 [M+K]$^+$; found 303.1373, 565.2196, 581.1936.

Elemental analysis (%)**:** Calc. for $C_{33}H_{34}O_7$: C 73.1, H 6.3; found: C 73.1, H 6.7.

1.4 Synthesis of methyl 4-(((2*S*)-3-(bis(4-methoxyphenyl)(phenyl)methoxy)-2-(((2-cyanoethoxy)(diisopropylamino)phosphaneyl)oxy)propoxy)methyl)benzoate (*S*)-6

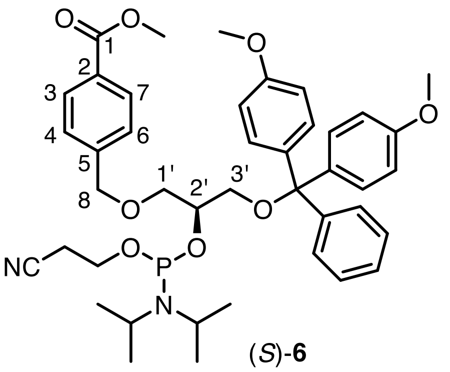

(*S*)-**6**

To a solution of compound (*S*)-**5** (255 mg, 0.470 mmol, 1.0 equiv.) and *N*,*N*-diisopropylethylamine (112 µL, 0.705 mmol, 1.5 equiv.) in dry dichloromethane (10 mL) was added 2-cyanoethyl N,N-diisopropylchlorophosphoramidite (126 µL, 0.564 mmol, 1.2 equiv.) dropwise and the reaction mixture was stirred for 2 h at room temperature. The solvent was removed under reduced pressure to afford the crude product (*S*)-**6** as a yellow oil. The compound was used for solid phase oligonucleotide synthesis without further purification.

$^{31}P\{^1H\}$ NMR (162 MHz, chloroform-*d*): δ = 149.4, 149.3.

HR-ESI MS (positive mode, MeCN)**:** *m/z* calc. for $C_{21}H_{19}O_2$ 303.1380 [DMT]$^+$, $C_{42}H_{52}N_2O_8P$ 743.3456 [M+H]$^+$, $C_{48}H_{67}N_3O_8P$ 844.4660 [M+$C_6H_{15}N$+H]$^+$; found 303.1378, 743.3453, 844.4657.

1.5 Synthesis of (*R*)-4-((2,3-dihydroxypropoxy)methyl)benzoic acid (*R*)-7

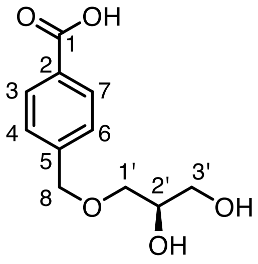

(*R*)-**7**

A solution of compound (*R*)-**4** (60 mg, 0.250 mmol, 1.0 equiv.) in 1 M aq. NaOH (5 mL) was stirred at 65 °C for 2.5 h. After neutralization with 1 M aq. HCl, the solvent was removed under reduced pressure and the obtained white solid was purified by column chromatography (dichloromethane/methanol, 10:1 to 5:1) to afford the product (*R*)-**7** as a white solid (51 mg, 0.225 mmol, 90%).

^{1}H NMR (600 MHz, methanol-*d*4): *δ* 8.00 (d, *J* = 8.3 Hz, 2H, 3-H and 7-H), 7.47 (d, *J* = 8.2 Hz, 2H, 4-H and 6-H), 4.63 (s, 2H, 8-H), 3.82 (p, *J* = 5.8 Hz, 1H, 2'-H), 3.63 – 3.50 (m, 4H, , 1'-H and , 3'-H).

^{13}C{^{1}H} NMR (151 MHz, methanol-*d*4): *δ* = 170.1 (C1), 144.9 (C5), 131.6 (C2), 130.8 (C3 and C7), 128.3 (C4 and C6), 73.7 (C8), 73.1 (C1'), 72.3 (C2'), 64.5 (C3').

The extinction coefficient at $\lambda = 260$ nm of (*R*)-**7** in water was determined to ε_{260} = 1111 L mol^{-1} cm^{-1}.

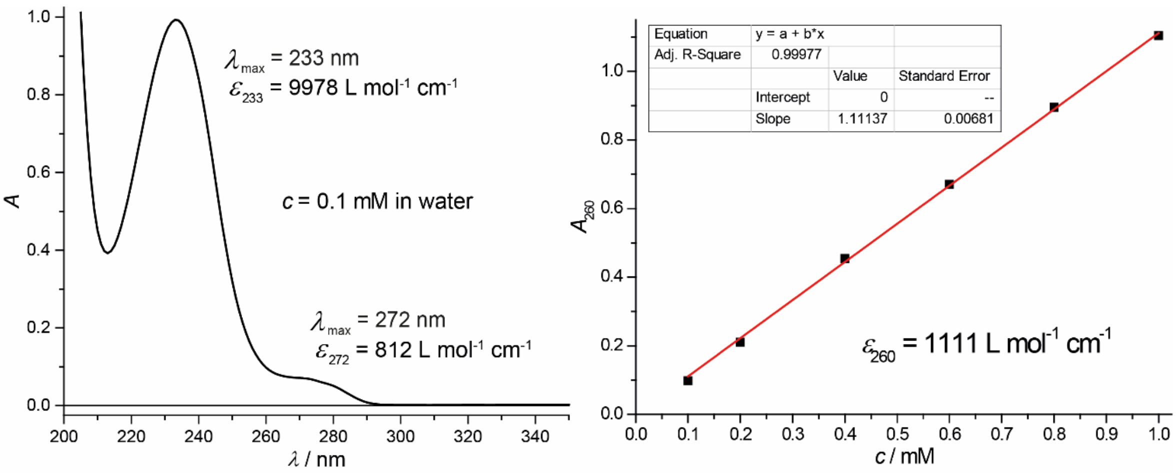

2 Single-Crystal X-ray Diffractional Analysis of (*R*)-4

Data collection was conducted on a *Bruker D8 Venture* four-circle diffractometer using a *PHOTON100* CMOS area detector. X-ray radiation was generated by microfocus source *IμS* Mo by *Incoatec* with *HELIOS* mirror optics and a *Bruker* single-hole collimator.

For the data collection, the *Bruker APEX 3 Suite* (v.2017.3-0) programs with the integrated programs *SAINT* (integration) and *SADABS* (adsorption correction) were used. Using *Olex*^{2} (Dolomanov et al., 2009), the structures were solved with the *ShelXT* (Sheldrick, 2015) structure solution program using Intrinsic Phasing and refined with the *XL* (Sheldrick, 2008) refinement package using Least Squares minimization. The absolute configuration was determined *via* anomalous dispersion.

Crystallographic data have been deposited at the CCDC (Cambridge Crystallographic Data Centre). These data can be obtained free of charge from the

CCDC, Union Road, Cambridge, CB2 IEZ, UK (Fax: +44-1223-336033; e-mail: deposit@ccdc.cam.ac.uk or www: http://www.ccdc.cam.ac.uk). CCDC deposition number 1961648.

Table S1: Crystal data and structure refinement of compound **(*R*)-4**.

Compound	**(*R*)-4**
Empirical formula	$C_{12}H_{16}O_5$
Formula weight	240.25
Temperature/K	100.0
Crystal system	monoclinic
Space group	$P2_1$
a/Å	4.6771(3)
b/Å	5.9336(3)
c/Å	20.7551(12)
α/°	90
β/°	93.4990(10)
γ/°	90
Volume/Å^3	574.92(6)
Z	2
ρ_{calc}g/cm^3	1.388
μ/mm^{-1}	0.908
F(000)	256.0
Crystal size/mm^3	0.495 × 0.407 × 0.296
Radiation	CuKα (λ = 1.54178)
2Θ range for data collection/°	4.266 to 144.992
Index ranges	-5 ≤ h ≤ 5, -7 ≤ k ≤ 7, -25 ≤ l ≤ 25
Reflections collected	10619
Independent reflections	2265 [R_{int} = 0.0214, R_{sigma} = 0.0182]
Data/restraints/parameters	2265/1/158
Goodness-of-fit on F^2	1.064
Final R indexes [I>=2σ (I)]	R_1 = 0.0223, wR_2 = 0.0584
Final R indexes [all data]	R_1 = 0.0224, wR_2 = 0.0592
Largest diff. peak/hole / e Å^{-3}	0.22/-0.13
Flack parameter	0.08(2)

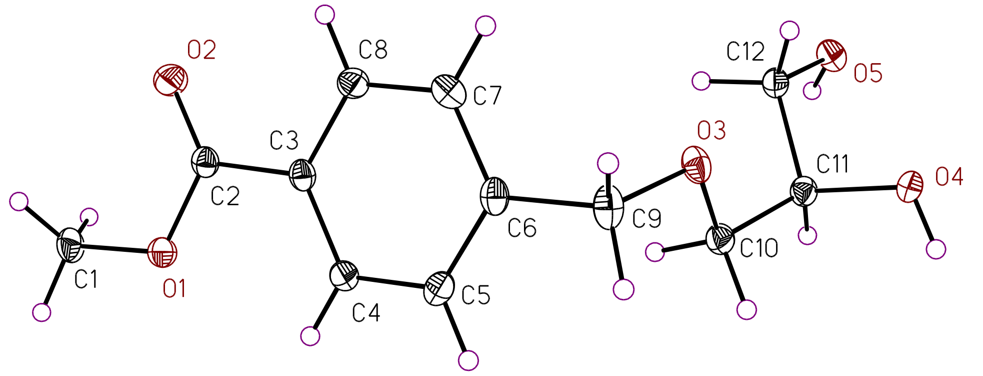

Fig. S1: Ortep (Farrugia, 1997) plot and numbering scheme of compound **(*R*)-4**. Displacement ellipsoids are drawn at 50% probability level.

3 Oligonucleotide Synthesis and Purification

All oligonucleotides were synthesized on a *K&A Laborgeraete GbR H-8* synthesizer on a 1 µmol scale using the standard phosphoramidite methods on CPG and following previously published procedures for synthesis (Engelhard et al., 2013; 2017).

Table 2: Reagents for DNA synthesis.

name	reagent	composition
DCA	detritylation	3% (v/v) dichloroacetic acid in anhydrous dichloromethane
ACT	activator	0.3 M 5-(benzylthio)-1H-tetrazole in anhydrous acetonitrile
Cap A	capping A	10% (v/v) N-methyl imidazole in anhydrous tetrahydrofurane
Cap B	capping B	2,6-lutidine / acetic anhydride / anhydrous tetrahydrofurane 1:1:8 (v/v/v)
OXI	Oxidizer	0.02 M iodine in tetrahydrofurane / pyridine / water 7:2:1 (v/v/v)
ACN		anhydrous acetonitrile

Standard phosphoramidites (DMT-dT-CEP, DMT-dA(Bz)-CEP and DMT-dG(*i*Bu)-CEP) were used and the cartridges were manually packed with controlled pore glass (CPG) solid supports (1000 Å, 25-35 µmol/g, DMT-dG(*i*Bu)-CPG). The oligonucleotide synthesis followed the built-in methods of the DNA synthesizer and was slightly modified. First, the cartridges were treated three times with DCA to deprotect the 5'-OH groups. Second, coupling was achieved by mixing the respective phosphoramidite (0.1 M in ACN) with ACT (1:1, v/v). The coupling time was ~0.5 min for standard phosphoramidites and ~3.5 min for the ligand-modified phosphoramidites. Third, the cartridge was treated with a 1:1 (v/v) mixture of Cap A and Cap B to acetylate unreacted 5'-OH groups, which was followed, by the oxidation with OXI. Here, an additional washing step with ACN was introduced compared to the standard routine. After each individual step of the cycle, the cartridge was washed with ACN followed by a drying step with argon. The described cycle was repeated for every incorporated nucleotide.

After DNA synthesis, the solid supports were removed from the cartridges and treated in 0.5 mL of 0.4 M NaOH in methanol/water (4:1) at 55 °C overnight for cleavage from the CPG and for deprotection. The supernatant solution was filtered (*VWR Centrifugal filters*) and the solid support was washed with 100 µL H_2O. The filtrate was diluted with water to a volume of 2 mL, methanol was removed under reduced pressure using a *H. Saur Laborbedarf S-Concentrator BA-VC-300H* vacuum concentrator and the volume of the solution was reduced to ~0.5 mL. 1.5 mL of 0.1 M TEAA buffer pH 7 were added, the sample was desalted using *Waters Sep-Pak* C18 cartridges and the volume was again reduced to ~0.3 mL using the vacuum concentrator. Purification of the oligonucleotides was performed with reversed-phase HPLC on an *Agilent Technologies 1260 Infinity II* HPLC system equipped with an autosampler, column oven, DAD detector and a *Macherey-Nagel VP 250/10 NUCLEODUR 100-5 C18ec* column (oven temperature: 60 °C, flow rate: 2.5 mL/min, solvent A: 50 mM TEAA pH 7, solvent B: 70:30 MeCN/50 mM TEAA pH 7, gradient: from 100% solvent A to 20% solvent A and 80% solvent B in 30 min).

Subsequently, the cleavage of the 5′-OH DMT protecting groups (with 2% TFA) and desalting were accomplished using *Waters Sep-Pak* C18 cartridges. Desalted oligonucleotides were lyophilized using a *Christ Alpha 2-4 LSCbasic* lyophilisation device and stored as a solid or as 0.2–1 mM stock solution in water at –20 °C.

The concentrations of all oligonucleotide stock solutions were determined *via* the absorbance at 260 nm at 25 °C with a *Thermo Scientific Nanodrop One* instrument and using extinction coefficients for the nucleosides (chapter 5.1 and Punt and Clever, 2019).

4 Analytics of Oligonucleotides

4.1 Analytical RP-HPLC

To check the purity of the synthesized and purified oligonucleotides, samples (10 µL) with DNA concentrations of around 500 µM in 20 mM TMAA pH 7 or TEAA pH 7 were prepared and analytical RP-HPLC was performed on an *Agilent Technologies 1260 Infinity II* system equipped with an autosampler, column oven, DAD detector and a *Macherey-Nagel EC 250/4.6 NUCLEODUR 100-5 C18ec* column (oven temperature: 60 °C, flow rate: 0.75 mL/min or 1.00 mL/min, solvent A: 50 mM TEAA pH 7, solvent B: 70:30 MeCN/50 mM TEAA pH 7). The RP-HPLC traces including the used solvent gradient are shown below.

L^{B}G3

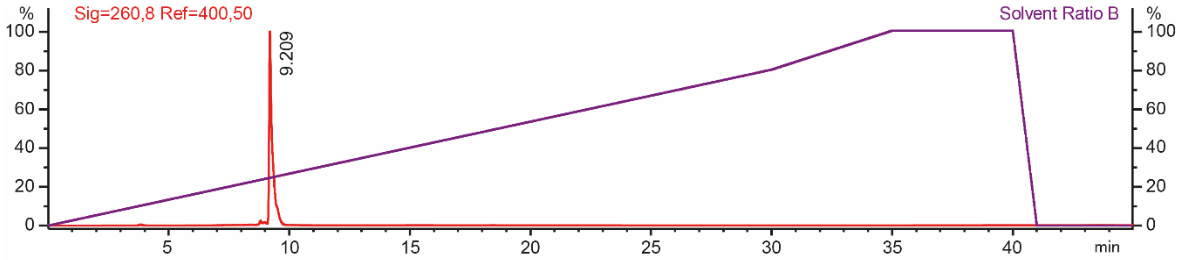

L^{B}G4

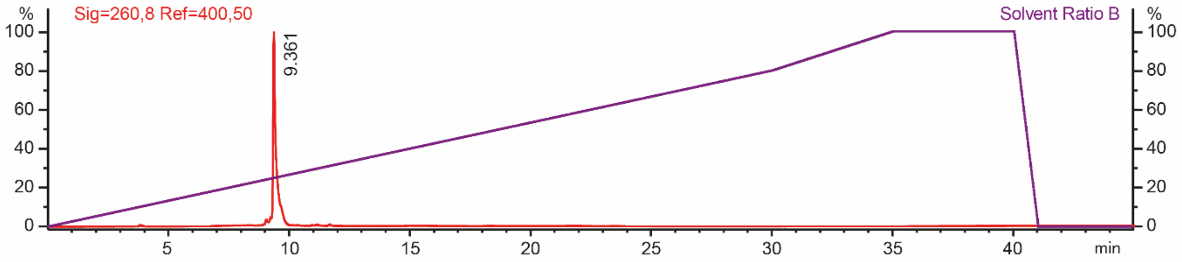

$\mathbf{L}^{\mathbf{B}}G_5$

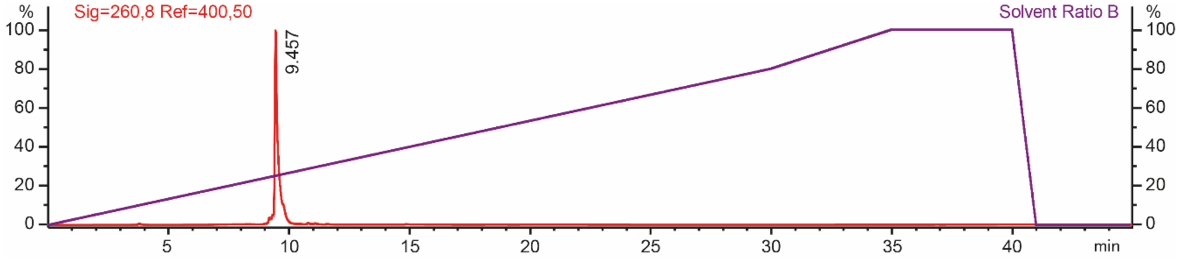

htel$\mathbf{L}^{\mathbf{B}}_4$

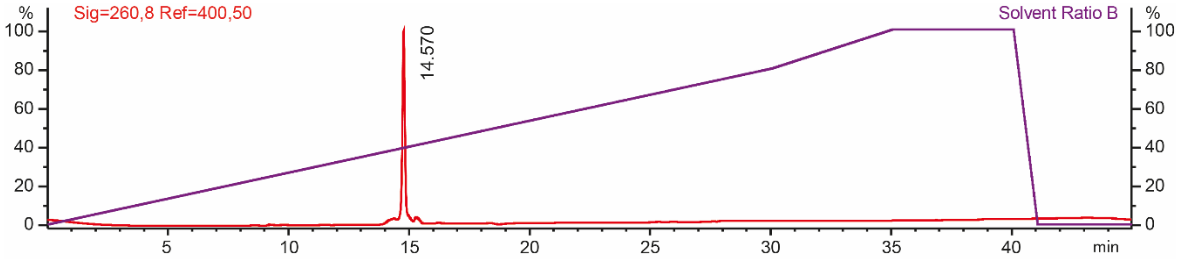

htel$\mathbf{L}^{\mathbf{B}}_3\mathbf{L}^{\mathbf{I}}$

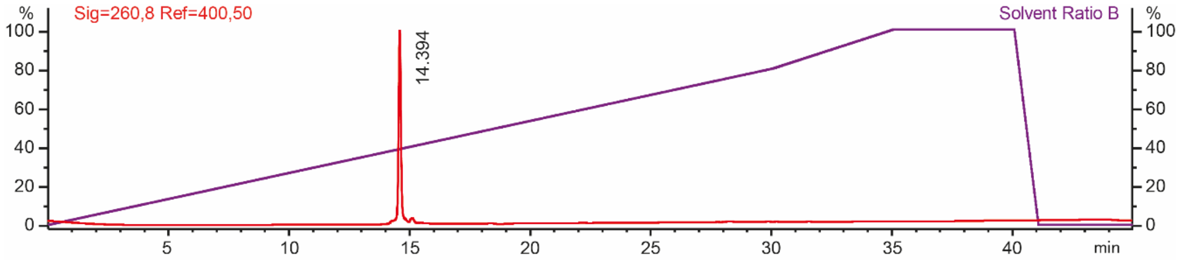

htel$\mathbf{L}^{\mathbf{B}}_2\mathbf{L}^{\mathbf{I}}_2$

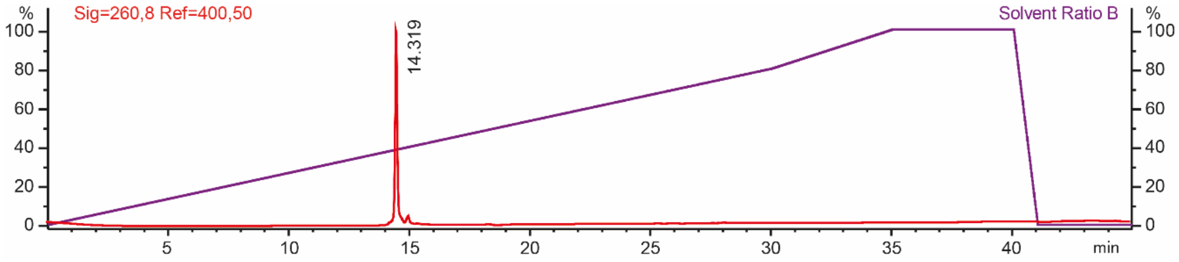

htel$\mathbf{L}^{\mathbf{B}}\mathbf{L}^{\mathbf{I}}_3$

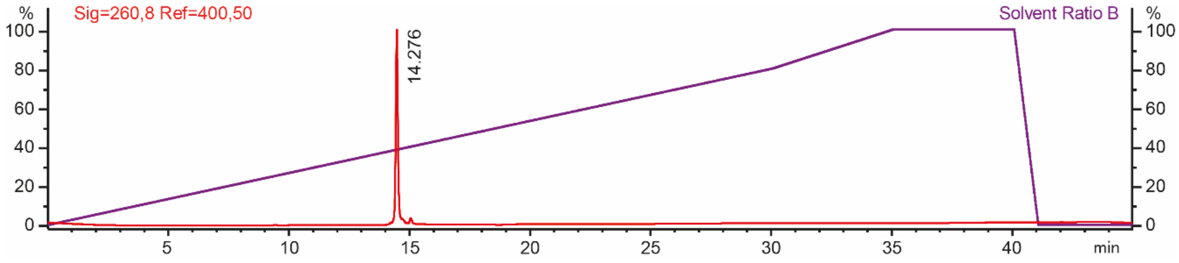

htel$\mathbf{L}^{\mathbf{B}}{}_2\mathbf{L}^{\mathbf{I}}{}_4$

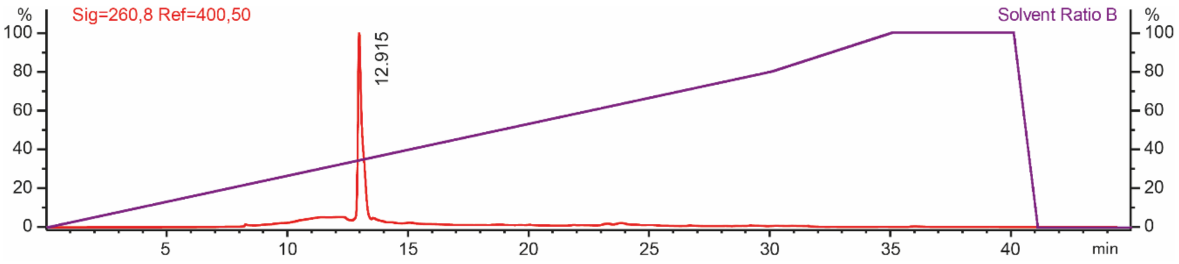

htel$\mathbf{L}^{\mathbf{B}}{}_4\mathbf{L}^{\mathbf{I}}{}_2$

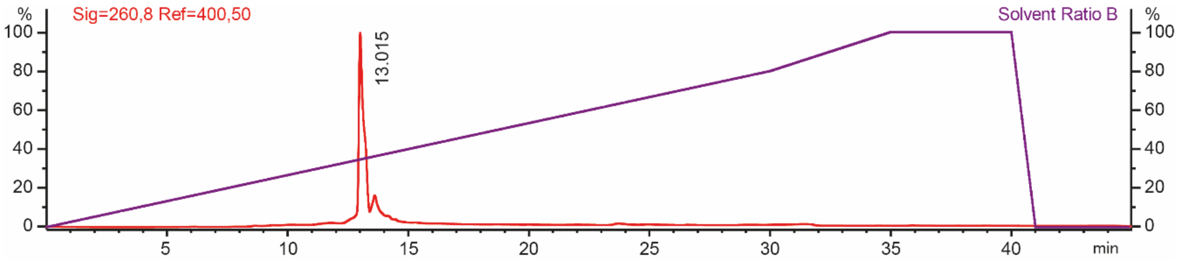

htel$\mathbf{L}^{\mathbf{B}}{}_3\mathbf{L}^{\mathbf{I}}{}_3$

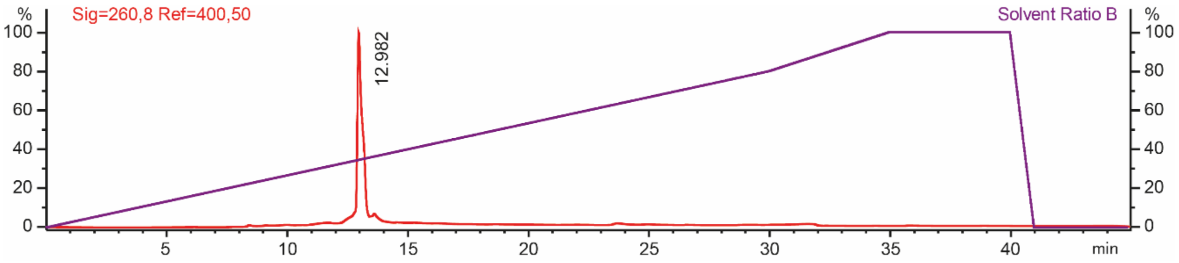

4.1 Mass Spectrometry

The quality of oligonucleotides was analyzed by mass spectrometry. For short oligos (<10 nt), MADLI-TOF MS was performed on a *Bruker ultrafleXtreme* MALDI-MS system (negative mode) with a 3-HPA-based matrix. Mass spectra are shown below.

L^B^G3

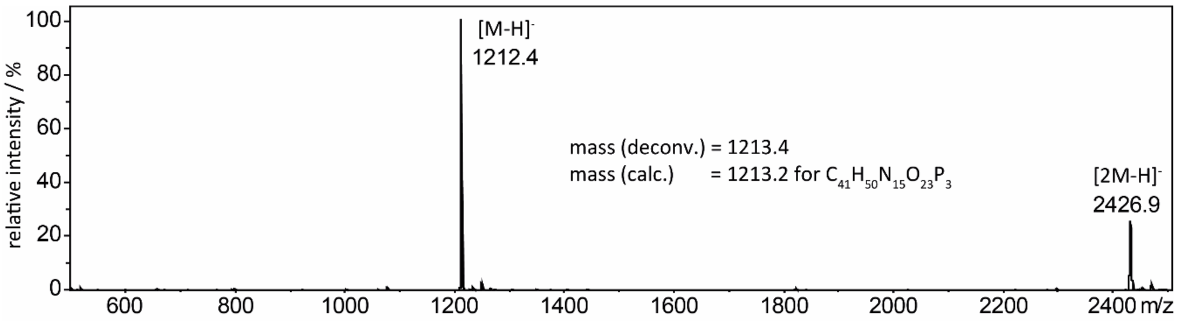

L^B^G4

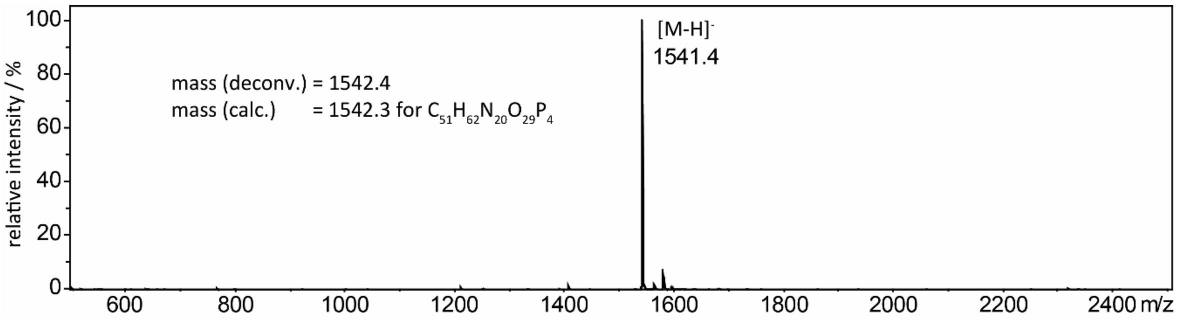

L^B^G5

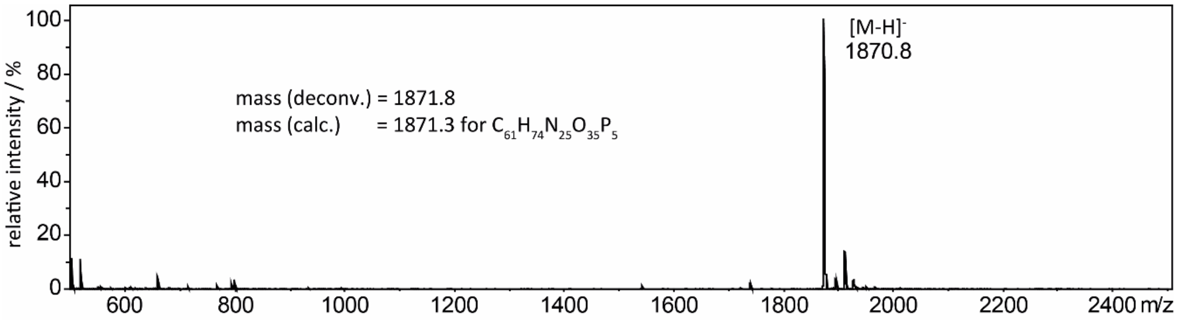

For longer oligos (>20 nt), samples (5 µL) with DNA concentrations of 150–300 µM in 15 mM TMAA pH 7 or TEAA pH 7 were prepared. ESI mass spectrometry was then performed on a *Bruker ESI-timsTOF* mass spectrometer (negative mode). For calibration of the TOF device, *Agilent ESI-Low Concentration Tuning Mix* was used. Automatic injection of the samples was achieved with the autosampler of an *Agilent Technologies 1260 Infinity* system (flow rate: 0.3 mL/min, solvent: MeCN/H_2O 1:1). The ESI mass spectra are shown below.

htel$\mathbf{L^B}_4$

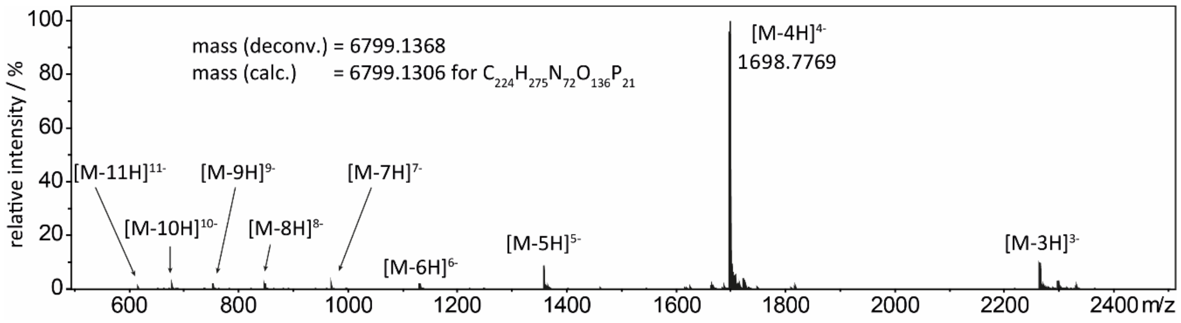

htel$\mathbf{L^I}_4$B

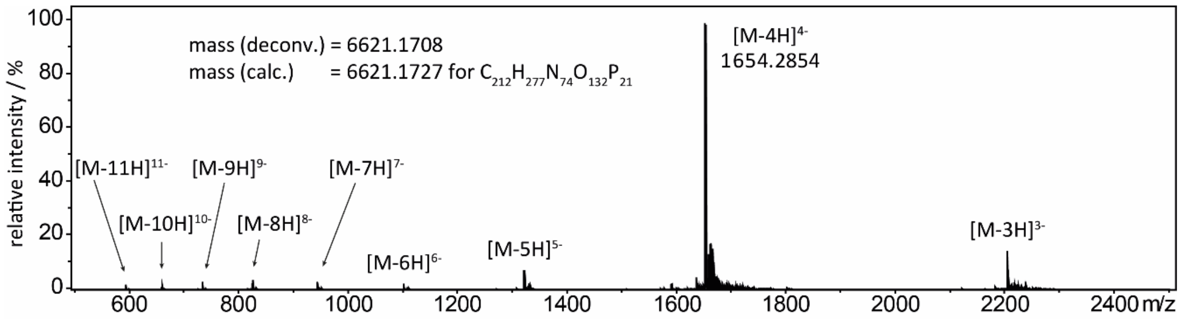

htel$\mathbf{L^B}_3\mathbf{L^I}$

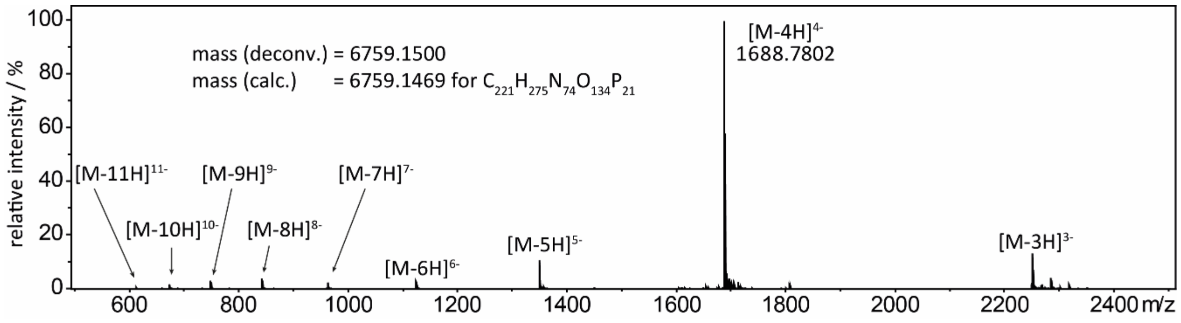

htel$\mathbf{L^B}_2\mathbf{L^I}_2$

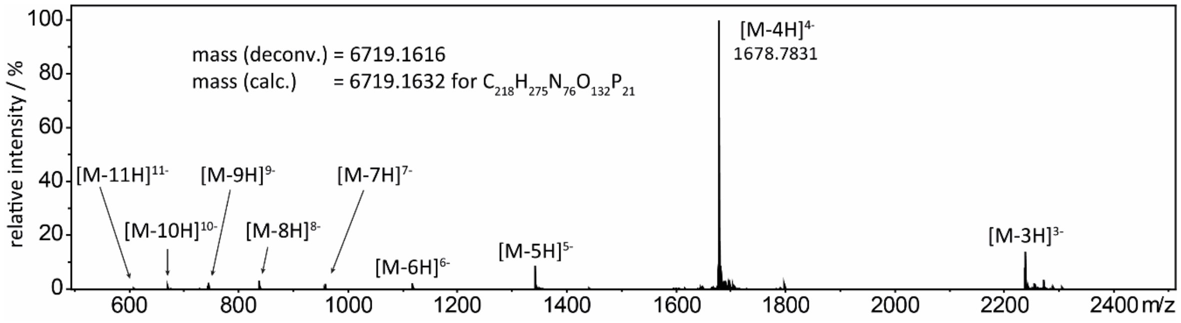

htelL^B L^I_3

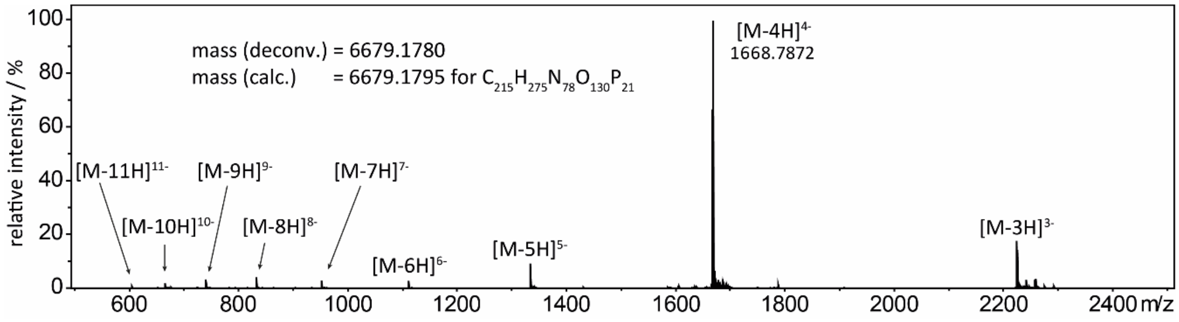

htelL^B_2 L^I_4

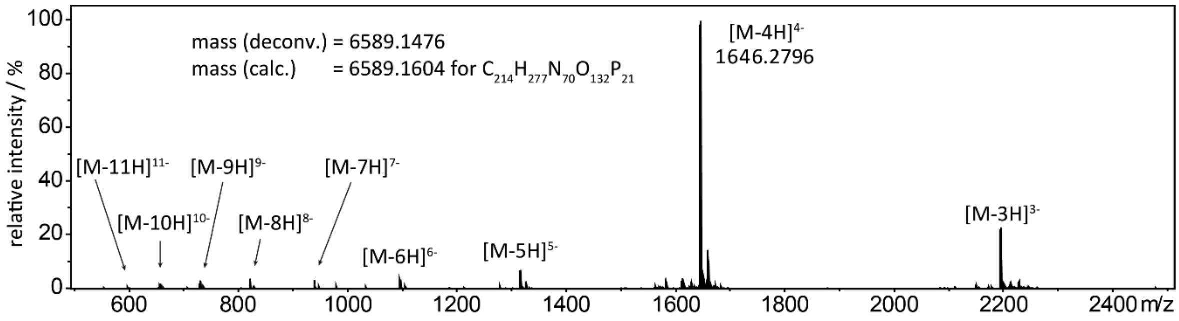

htelL^B_4 L^I_2

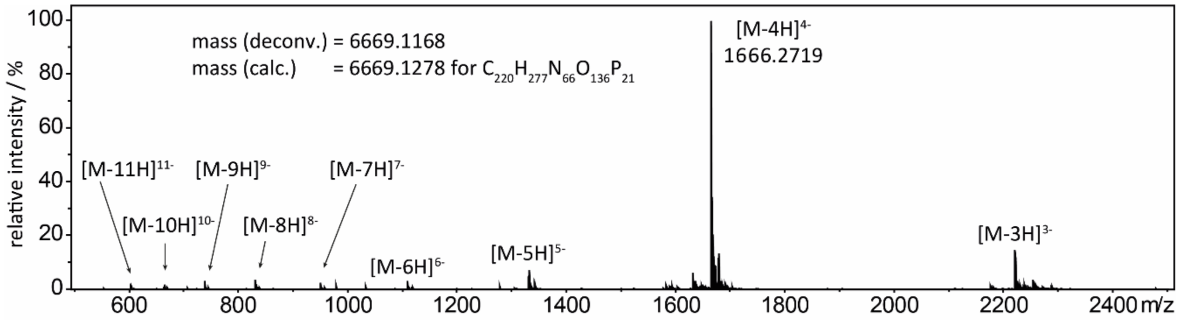

htelL^B_3 L^I_3

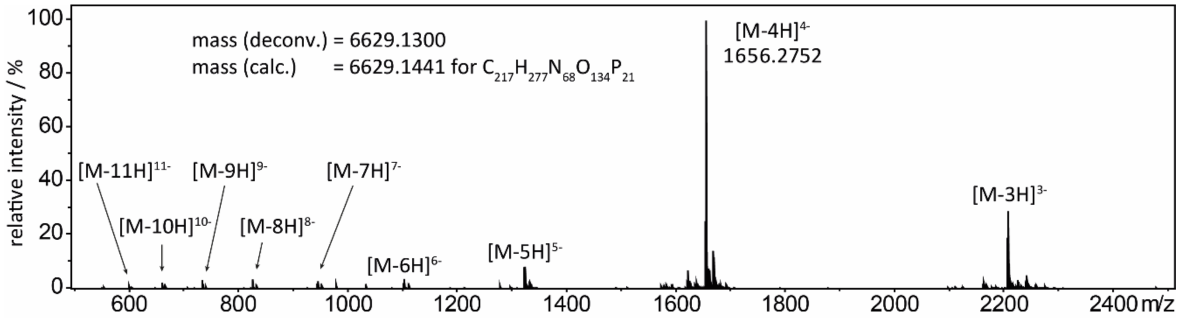

5 UV-based Thermal Denaturation Studies

5.1 Sample Preparation

For all UV-based thermal denaturation studies, tetramolecular G-quadruplex samples contained 4 µM single-stranded DNA, 100 mM NaCl, 10 mM lithium cacodylate buffer pH 7.2 and, if present, 1, 2, 3 or 5 µM $CuSO_4$ or 1 µM $NiSO_4$, ZnI_2, $GdCl_3$ or $CeCl_3$.

Unimolecular G-quadruplex samples contained 1.88 µM single-stranded DNA, 100 mM KCl, 10 mM lithium cacodylate buffer pH 7.2 and, if present, 1.88 or 3.75 µM $Co(NO_3)_2$, $NiSO_4$, $CuSO_4$, ZnI_2 or $VOSO_4$.

For all experiments, samples were prepared with ultrapure water (type I, 18.2 MΩ cm), obtained with a *VWR Puranity TU 3 UV*.

Samples were heated to 85 °C for 10 min, slowly cooled to 4 °C with a cooling rate of 0.5 °C/min and then left at this temperature for several hours (typically overnight). To ensure full formation of tetramolecular G-quadruplexes, the respective samples were frozen at –20 °C for 1 h and thawed again to 4 °C (Zhai et al., 2012).

5.2 Spectrometer and Methods

Both UV spectra and thermal denaturation profiles (melting curves) were recorded on a *Jasco V-650* or *Jasco V-750 UV-Visible* Spectrophotometer equipped with a *PAC-743 6-cell thermostat* for temperature control. The temperature was measured in the measurement cell in a water-filled cuvette. Quartz glass cuvettes (*Hellma Analytics114-QS*, 1 cm path length) were used. In order to avoid condensation of water on the cuvette surface or cell window at low temperatures, a constant flow of dried air was pumped through the measurement cell. Evaporation of water at high temperatures and resulting changes in the absorption behaviour were minimized by a thin layer of silicon oil placed onto the sample and by tightly stoppering the cuvette.

UV spectra were recorded from 350 to 220 nm with a scan rate of 200 nm/min both before (0 or 4 °C) and after thermal denaturation (85 °C). The data interval was set to 1 nm, bandwidth to 2.0 nm and the response time to 0.96 sec. To obtain the thermal difference spectra (TDS), the spectrum before denaturation (at 0 or 4 °C) was subtracted from the one after denaturation (at 85 °C). The resulting

spectra was zeroed using the absorption at 350 nm. A negative band (hypochromic shift) at 295±2 nm and positive bands at 243±2 nm and 273±2 nm (hyperchromic shift) indicated G-quadruplex formation (Mergny et al., 2005).

For the thermal denaturation profiles (melting curves), absorption of the samples at 295 nm was recorded in a 0.5 °C interval with a temperature gradient set to 0.5 °C/min, which corresponds to ~0.174 °C/min including the measurement time. To prevent major deviations from this temperature gradient, the absorption of always five cuvettes was measured. Data points were recorded from 0 or 4 °C to 85 °C and melting curves were background corrected using the absorption at 350 nm. If possible (when denaturation temperatures were >20 °C), melting curves were converted to the fraction folded values by linear fitting of the low and high temperature baselines (Mergny and Lacroix, 2009). Thermal denaturation temperatures were then determined by reading the respective value at the fraction folded value α = 0.5. For melting curves with denaturation temperatures <20 °C, the thermal denaturation temperatures were determined by calculating the first derivative and reading the temperature value at its minimum.

Note that for unimolecular G-quadruplexes at every temperature an equilibrium between folded and unfolded species is given. This is not true for tetramolecular G-quadruplexes which is the reason why the melting temperature for tetramolecular G-quadruplexes depends on the heating rate.

5.3 Thermal Denaturation Profiles and Thermal Difference Spectra

L$^{\mathbf{B}}$G$_3$

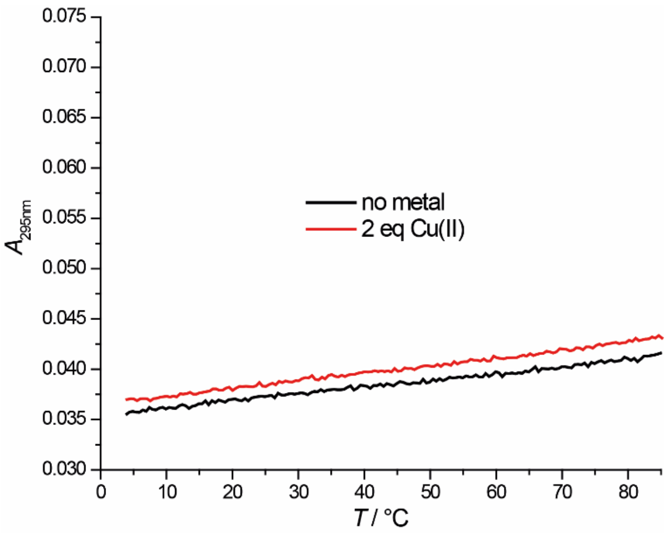

Fig. S2: Temperature-dependent absorption of **L**$^{\mathbf{B}}$G$_3$ at 295 nm. No thermal denaturation is observed in absence or presence of 2 equiv. $CuSO_4$, which suggests no G-quadruplex formation.

L$^{\mathbf{B}}$G$_4$

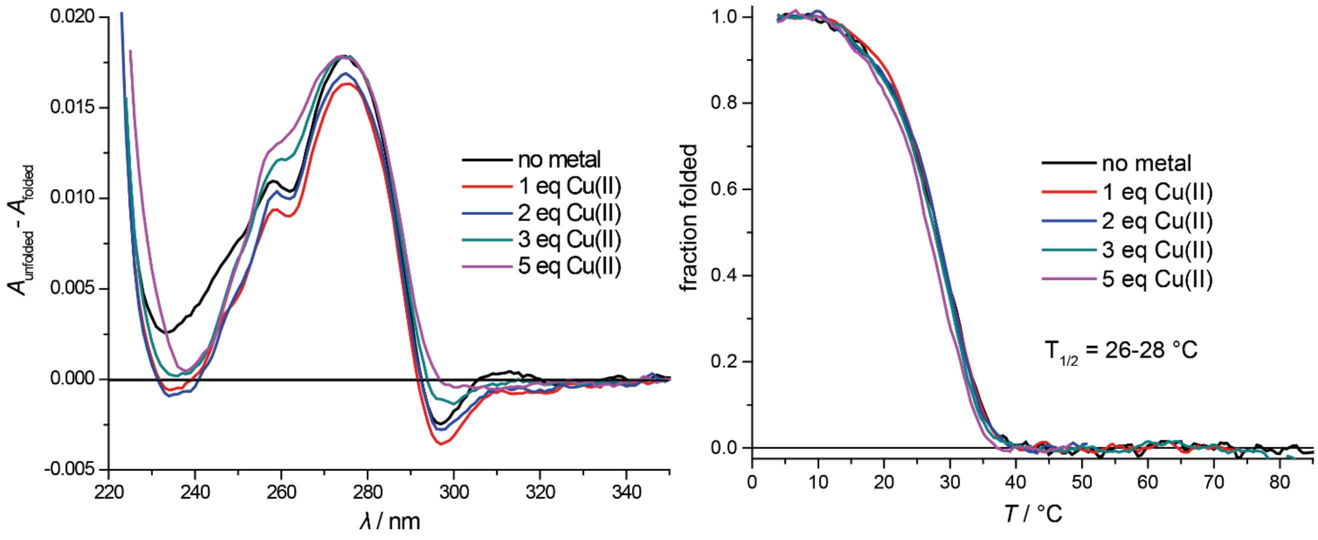

Fig. S3: Thermal difference spectra (left) and thermal denaturation profiles (right) of G-quadruplex (**L**$^{\mathbf{B}}$G$_4$)$_4$ in absence or presence of 1, 2, 3 or 5 equiv. $CuSO_4$. No thermal stabilization could be observed after addition of Cu(II).

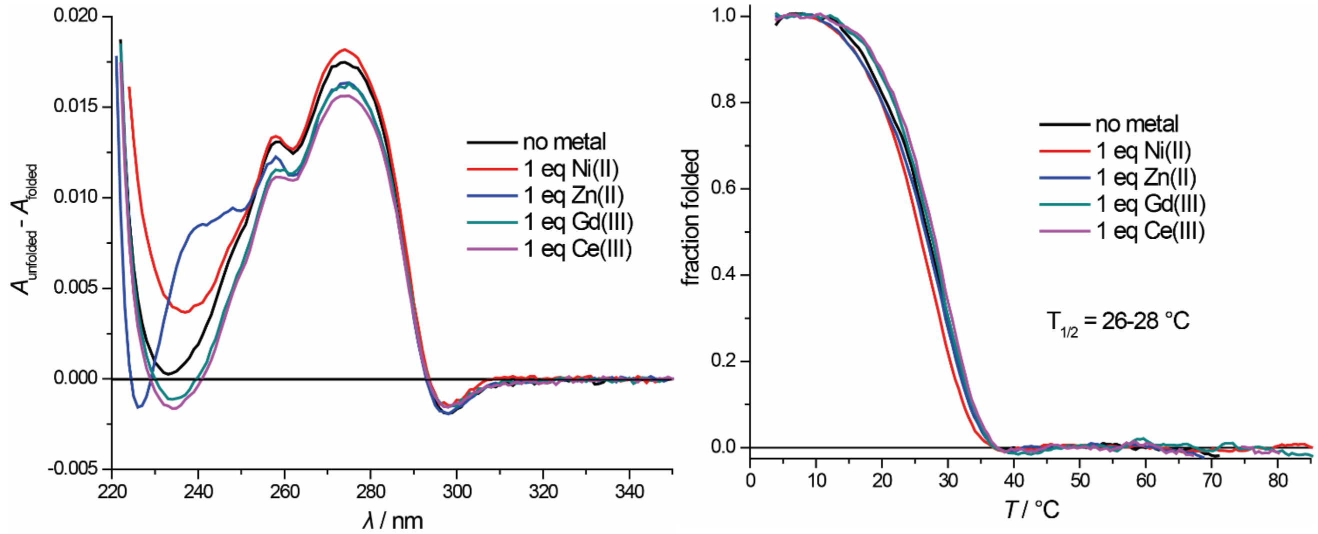

Fig. S4: Thermal difference spectra (left) and thermal denaturation profiles (right) of G-quadruplex (**L**$^{\mathbf{B}}$G$_4$)$_4$ in absence or presence of 1 equiv. $NiSO_4$, ZnI_2, $GdCl_3$ or $CeCl_3$. No thermal stabilization could be observed after addition of transition metal ions.

L^{B}G$_{5}$

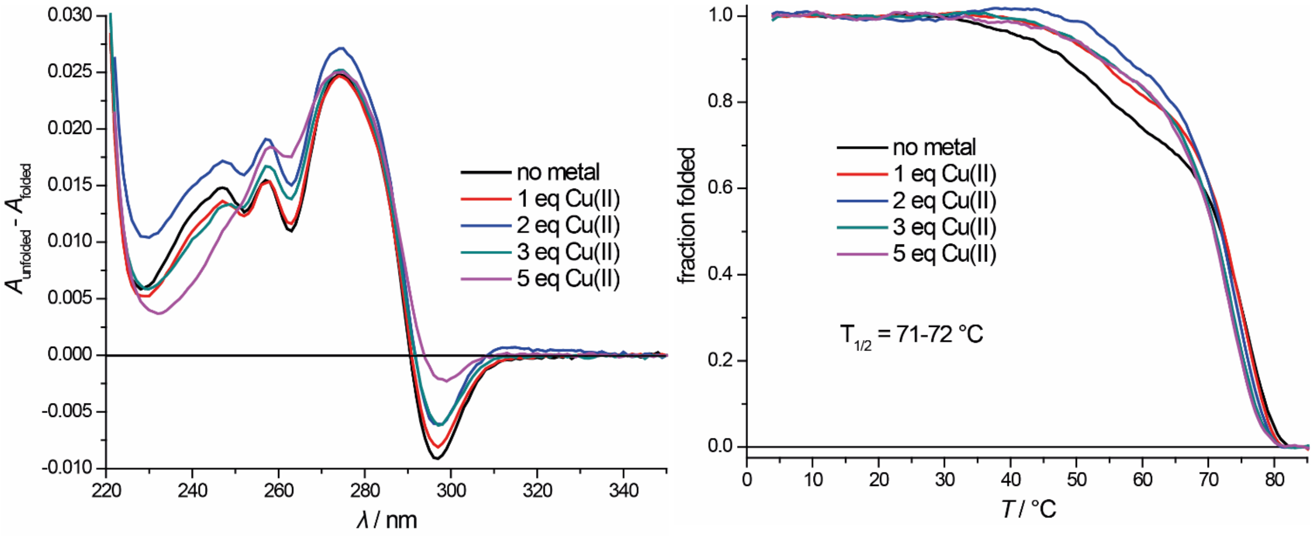

Fig. S5: Thermal difference spectra (left) and thermal denaturation profiles (right) of G-quadruplex (**L**^{B}G$_{5}$)$_{4}$ in absence or presence of 1, 2, 3 or 5 equiv. $CuSO_4$. No thermal stabilization could be observed after addition of Cu(II).

htel**L**$^{\mathbf{B}}$$_{\mathbf{4}}$

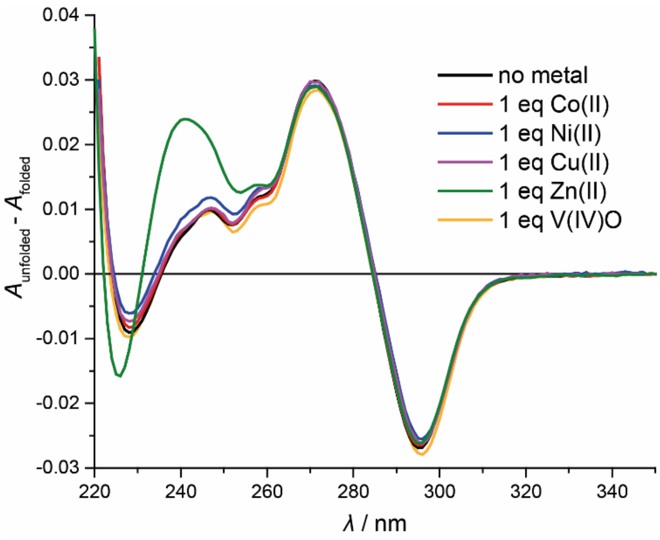

Fig. S6: Thermal difference spectra of G-quadruplex htel**L**$^{\mathbf{B}}$$_{\mathbf{4}}$ in absence or presence of 1 equiv. $Co(NO_3)_2$, $NiSO_4$, $CuSO_4$, ZnI_2 or $VOSO_4$.

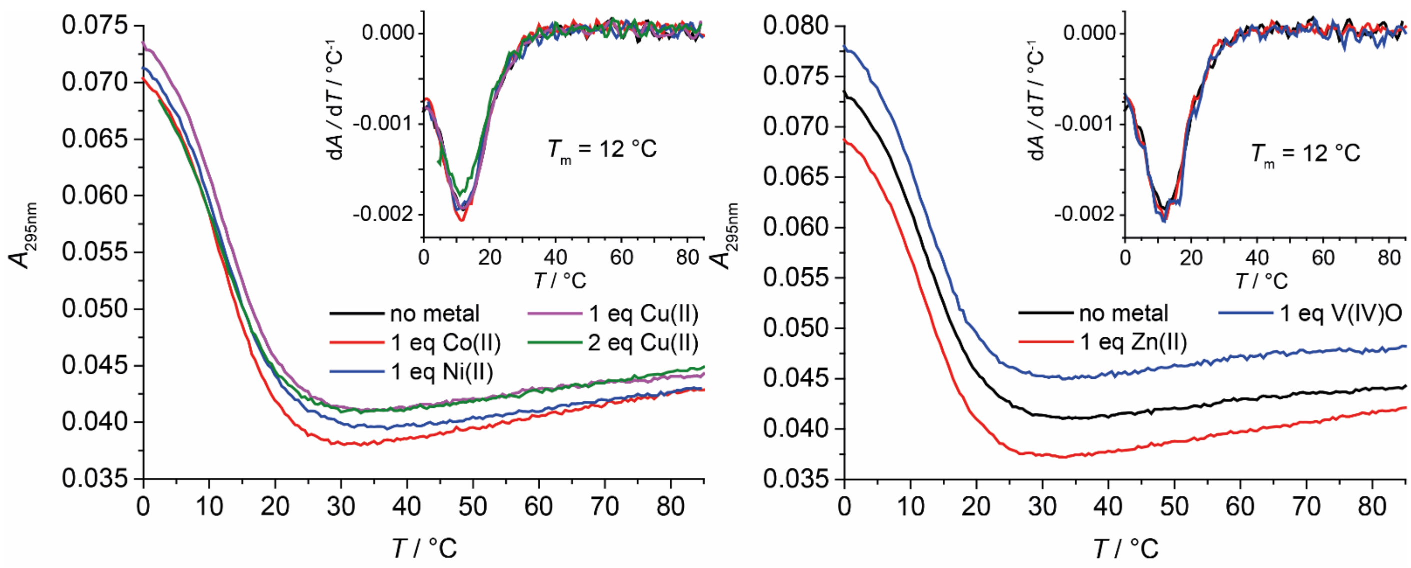

Fig. S7: Thermal denaturation profiles of G-quadruplex htel$\mathbf{L}^{\mathbf{B}}_{\mathbf{4}}$ in absence or presence of 1 equiv. $Co(NO_3)_2$, $NiSO_4$, ZnI_2 or $VOSO_4$ or of 1 or 2 equiv. $CuSO_4$. No thermal stabilization could be observed after addition of transition metal ions.

htel$\mathbf{L}^{\mathbf{I}}_4$B

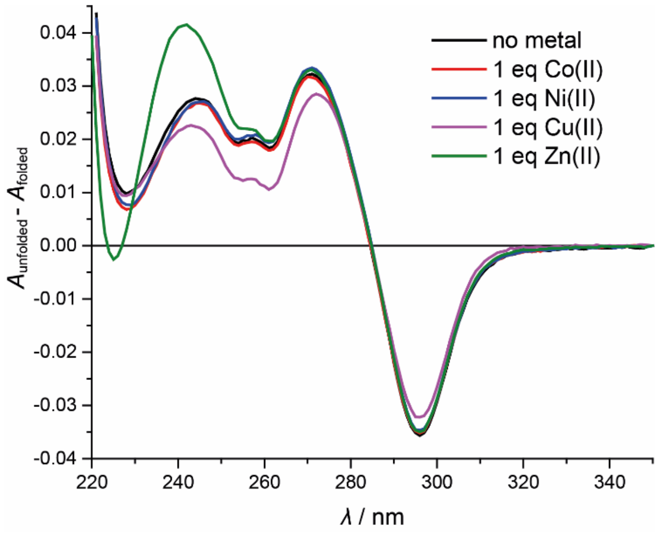

Fig. S8: Thermal difference spectra of G-quadruplex htel$\mathbf{L}^{\mathbf{I}}_{\mathbf{4}}$B in absence or presence of 1 equiv. $Co(NO_3)_2$, $NiSO_4$, $CuSO_4$ or ZnI_2.

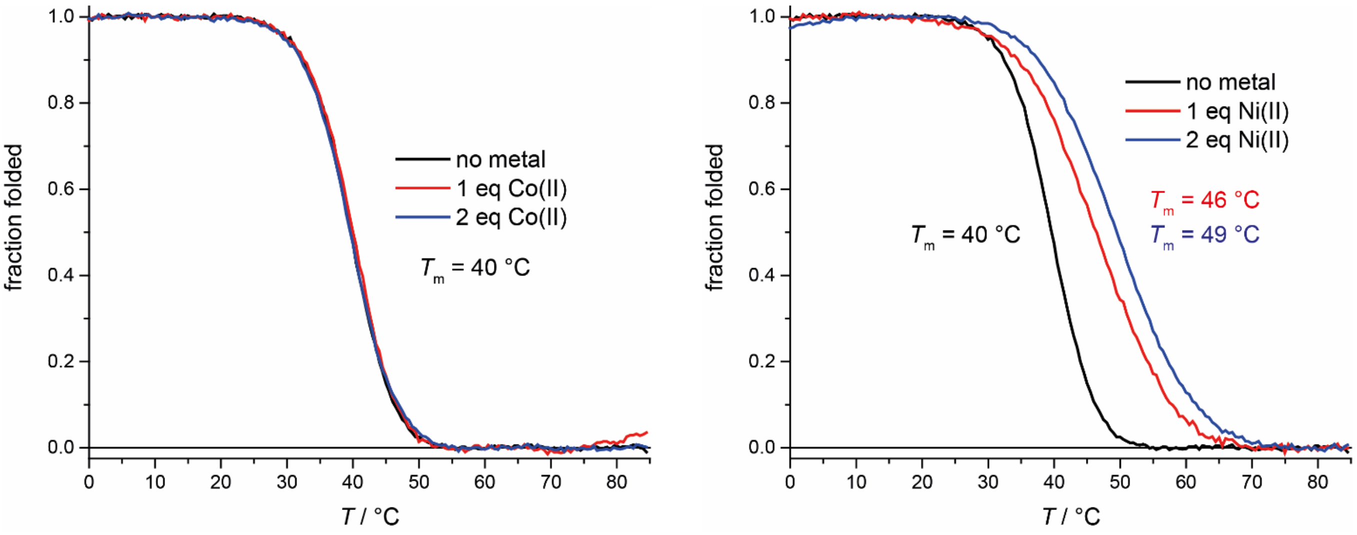

Fig. S9: Thermal denaturation profiles of G-quadruplex htel$\mathbf{L}^{\mathrm{I}}_{4}$B in absence or presence of 1 or 2 equiv. $Co(NO_3)_2$ or $NiSO_4$. No thermal stabilization could be observed after addition of Co(II) whereas a thermal stabilization of ΔT_m = 6 °C could be observed after addition of Ni(II).

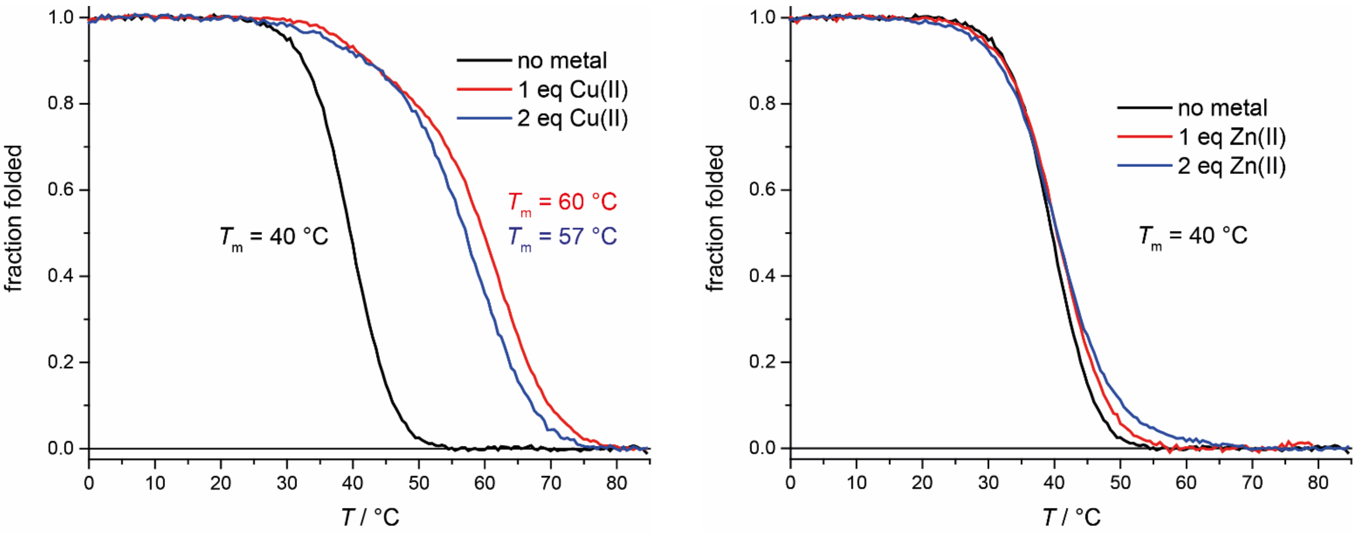

Fig. S10: Thermal denaturation profiles of G-quadruplex htel$\mathbf{L}^{\mathrm{I}}_{4}$B in absence or presence of 1 or 2 equiv. $CuSO_4$ or ZnI_2. A thermal stabilization of ΔT_m = 20 °C could be observed after addition of Cu(II) whereas no thermal stabilization could be observed after addition of Zn(II).

htel$L^B{}_3L^I$

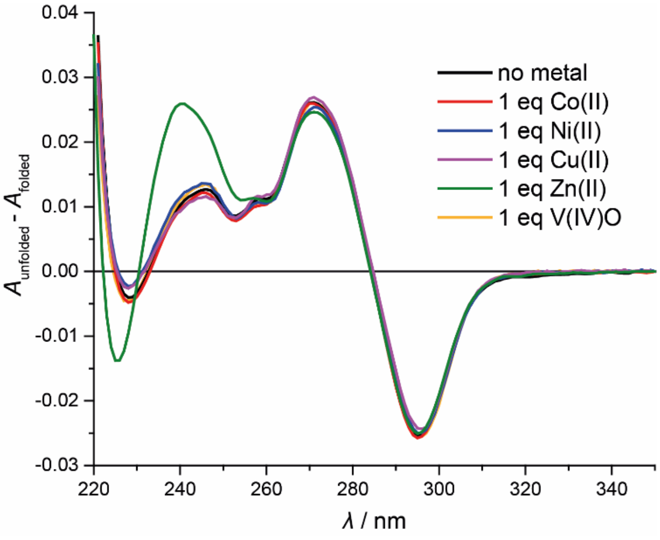

Fig. S11: Thermal difference spectra of G-quadruplex htel$L^B{}_3L^I$ in absence or presence of 1 equiv. of $Co(NO_3)_2$, $NiSO_4$, $CuSO_4$, ZnI_2 or $VOSO_4$.

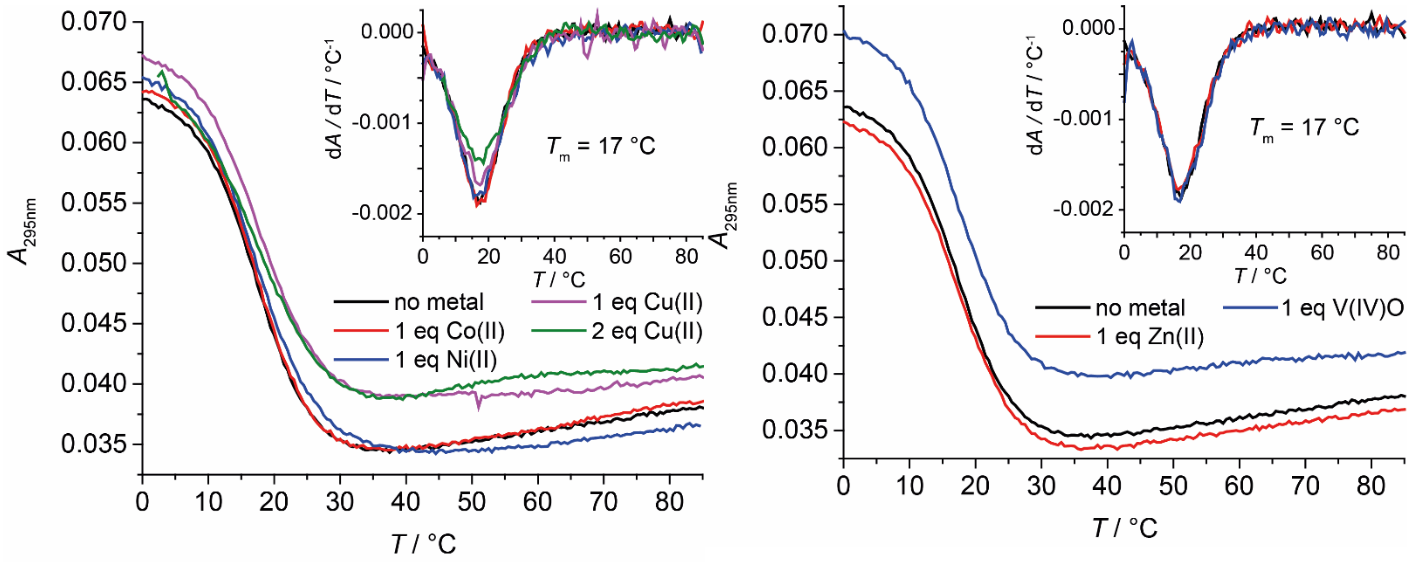

Fig. S12: Thermal denaturation profiles of G-quadruplex htel$L^B{}_3L^I$ in absence or presence of 1 equiv. $Co(NO_3)_2$, $NiSO_4$, ZnI_2 or $VOSO_4$ or of 1 or 2 equiv. $CuSO_4$. No thermal stabilization could be observed after addition of transition metal ions.

htel$L^{B}_{2}L^{I}_{2}$

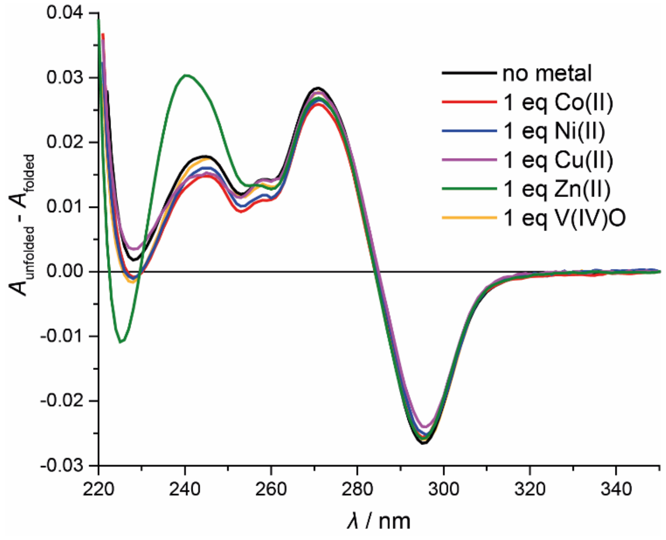

Fig. S13: Thermal difference spectra of G-quadruplex htel$L^{B}_{2}L^{I}_{2}$ in absence or presence of 1 equiv. of $Co(NO_3)_2$, $NiSO_4$, $CuSO_4$, ZnI_2 or $VOSO_4$.

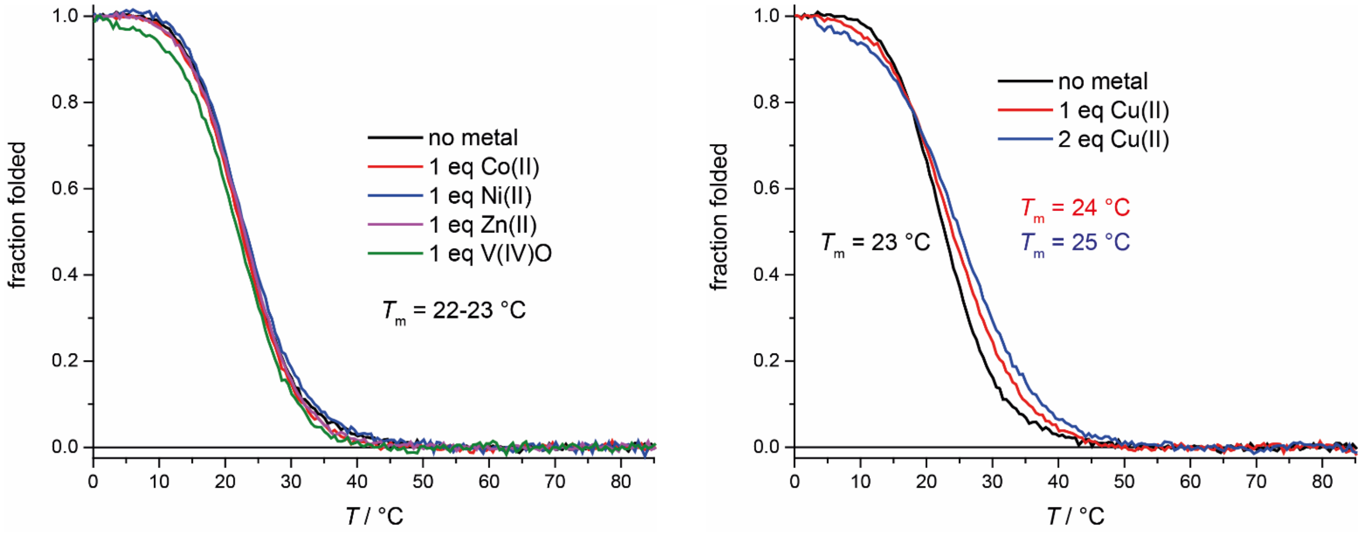

Fig. S14: Thermal denaturation profiles of G-quadruplex htel$\mathbf{L^{B}_{2}L^{I}_{2}}$ in absence or presence of 1 equiv. $Co(NO_3)_2$, $NiSO_4$, ZnI_2 or $VOSO_4$ or of 1 or 2 equiv. $CuSO_4$. A small thermal stabilization (ΔT_m = 1 °C) could only be observed after addition of Cu(II).

htel$\mathbf{L}^{\mathrm{B}}\mathbf{L}^{\mathrm{I}}_{3}$

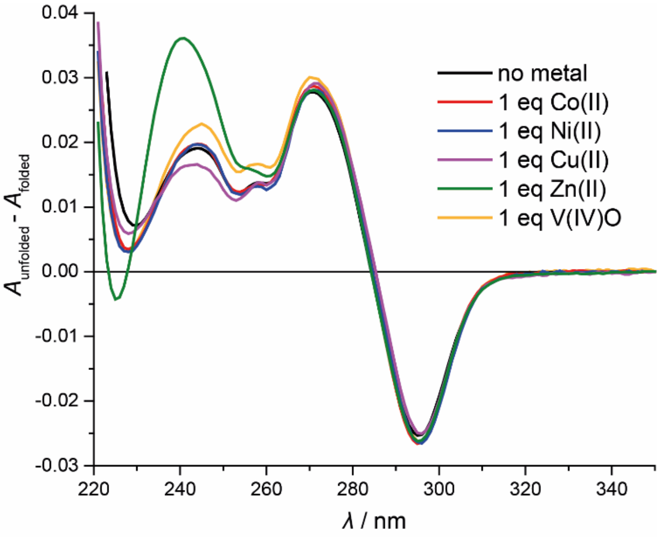

Fig. S15: Thermal difference spectra of G-quadruplex htel$\mathbf{L}^{\mathrm{B}}\mathbf{L}^{\mathrm{I}}_{3}$ in absence or presence of 1 equiv. of $Co(NO_3)_2$, $NiSO_4$, $CuSO_4$, ZnI_2 or $VOSO_4$.

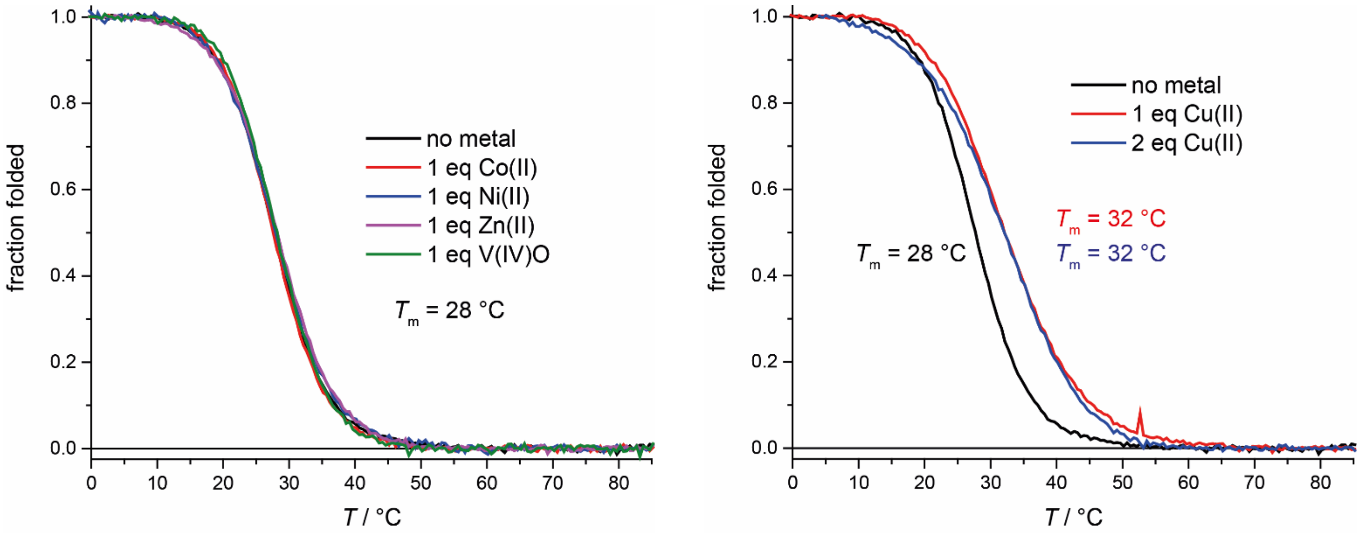

Fig. S16: Thermal denaturation profiles of G-quadruplex htel$\mathbf{L}^{\mathrm{B}}\mathbf{L}^{\mathrm{I}}_{3}$ in absence or presence of 1 equiv. $Co(NO_3)_2$, $NiSO_4$, ZnI_2 or $VOSO_4$ or of 1 or 2 equiv. $CuSO_4$. A thermal stabilization of ΔT_m = 4 °C could only be observed after addition of Cu(II).

htel$\mathbf{L}^{B}{}_{2}\mathbf{L}^{I}{}_{4}$

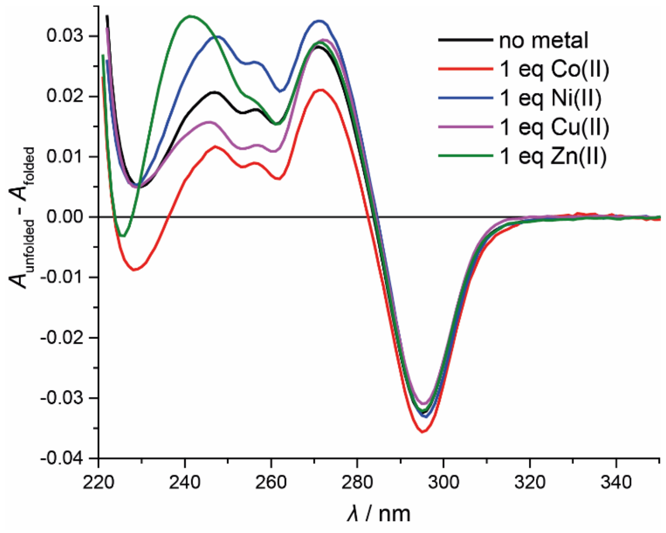

Fig. S17: Thermal difference spectra of G-quadruplex htel$\mathbf{L}^{B}{}_{2}\mathbf{L}^{I}{}_{4}$ in absence or presence of 1 equiv. $Co(NO_3)_2$, $NiSO_4$, $CuSO_4$ or ZnI_2.

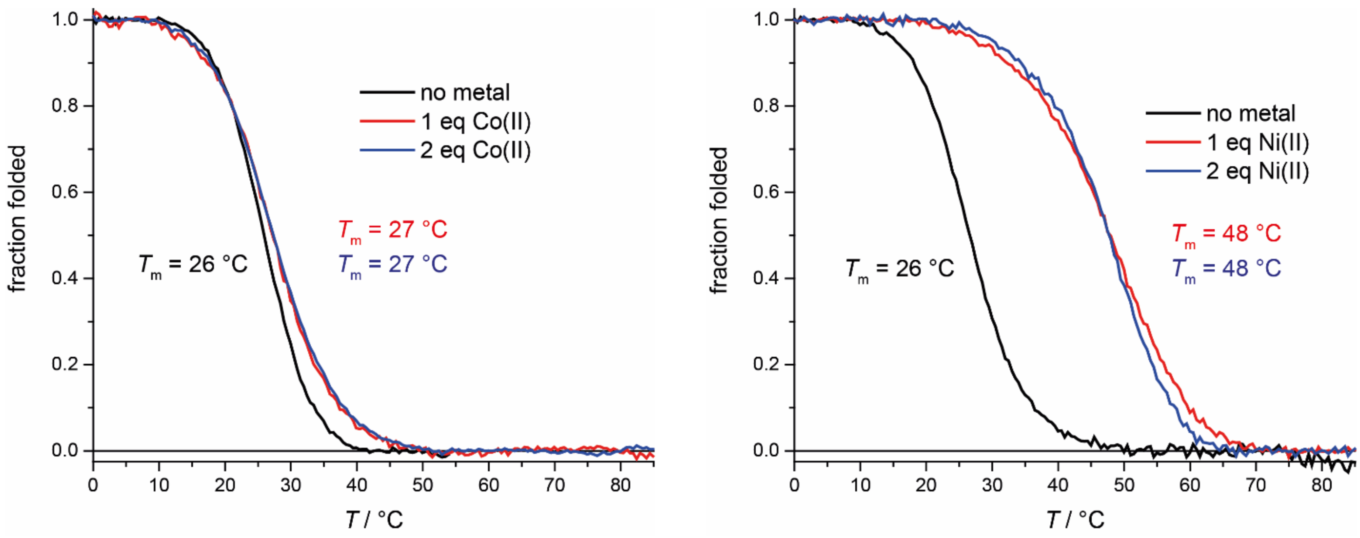

Fig. S18: Thermal denaturation profiles of G-quadruplex htel$\mathbf{L}^{B}{}_{2}\mathbf{L}^{I}{}_{4}$ in absence or presence of 1 or 2 equiv. $Co(NO_3)_2$ or $NiSO_4$. A small thermal stabilization of ΔT_m = 1 °C could be observed after addition of Co(II) whereas a strong stabilization of ΔT_m = 22 °C could be observed after addition of Ni(II).

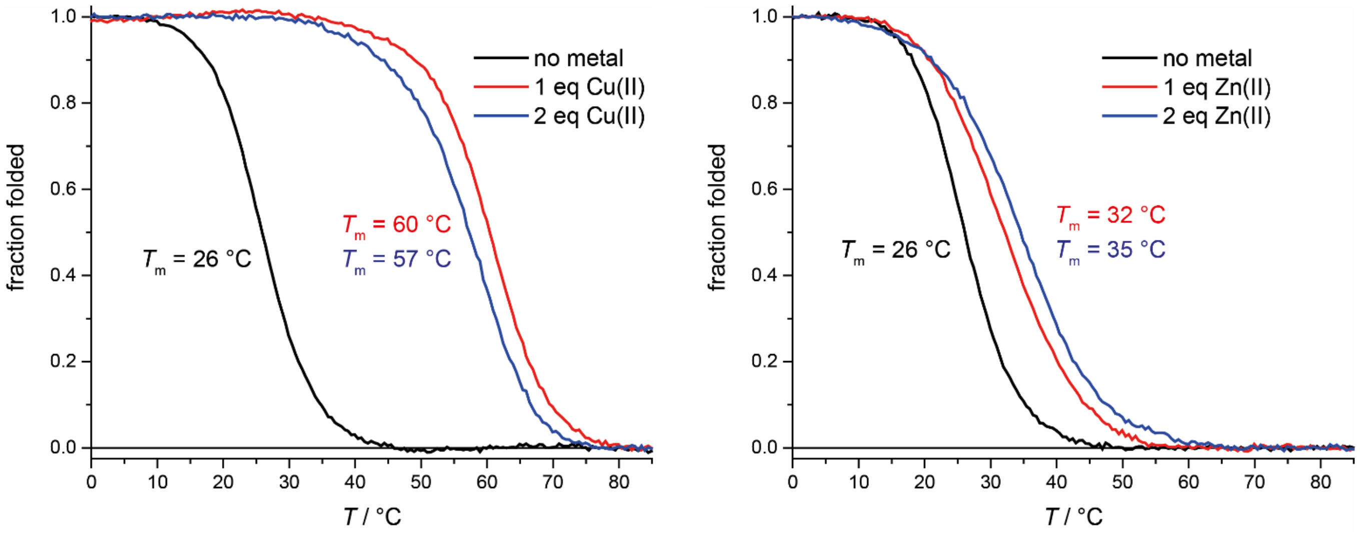

Fig. S19: Thermal denaturation profiles of G-quadruplex htel$\mathbf{L}^{B}_{2}\mathbf{L}^{I}_{4}$ in absence or presence of 1 or 2 equiv. $CuSO_4$ or ZnI_2. Thermal stabilizations of ΔT_m = 34 °C after addition of Cu(II) and of ΔT_m = 6 °C after addition of Zn(II) could be observed.

htel$\mathbf{L}^{B}_{4}\mathbf{L}^{I}_{2}$

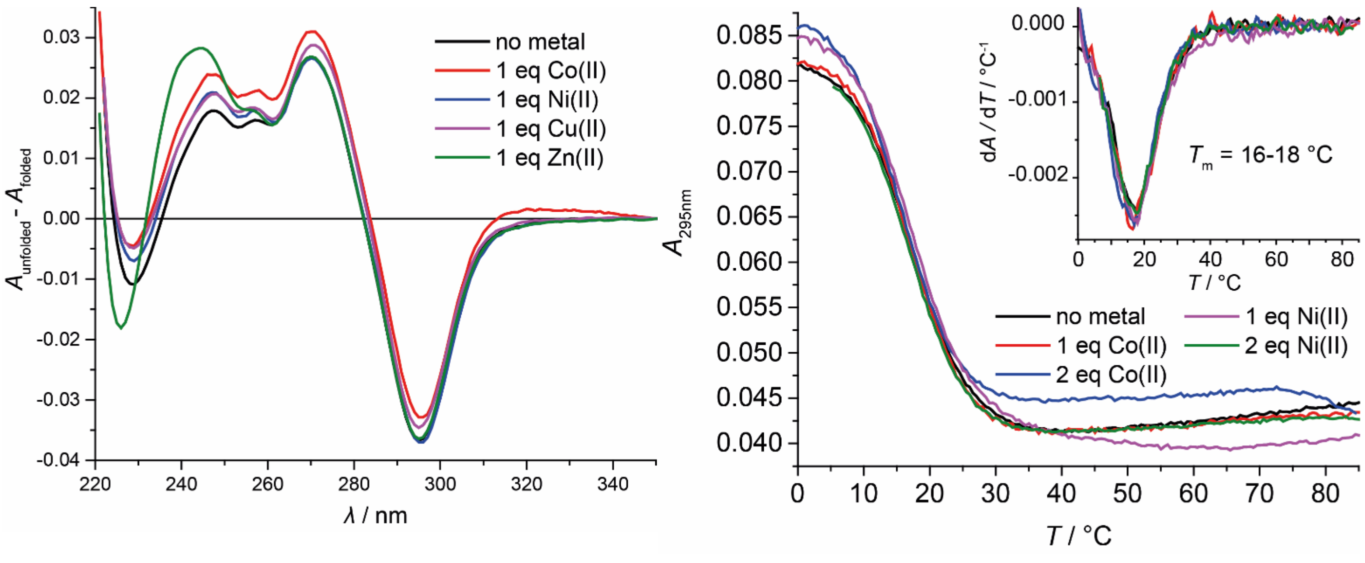

Fig. S20: Thermal difference spectra of G-quadruplex htel$\mathbf{L}^{B}_{4}\mathbf{L}^{I}_{2}$(left) in absence or presence of 1 equiv. $Co(NO_3)_2$, $NiSO_4$, $CuSO_4$ or ZnI_2. Thermal denaturation profiles of G-quadruplex htel$\mathbf{L}^{B}_{4}\mathbf{L}^{I}_{2}$ (right) in absence or presence of 1 or 2 equiv. $Co(NO_3)_2$ or $NiSO_4$. No thermal stabilization could be observed after addition of the transition metal ions.

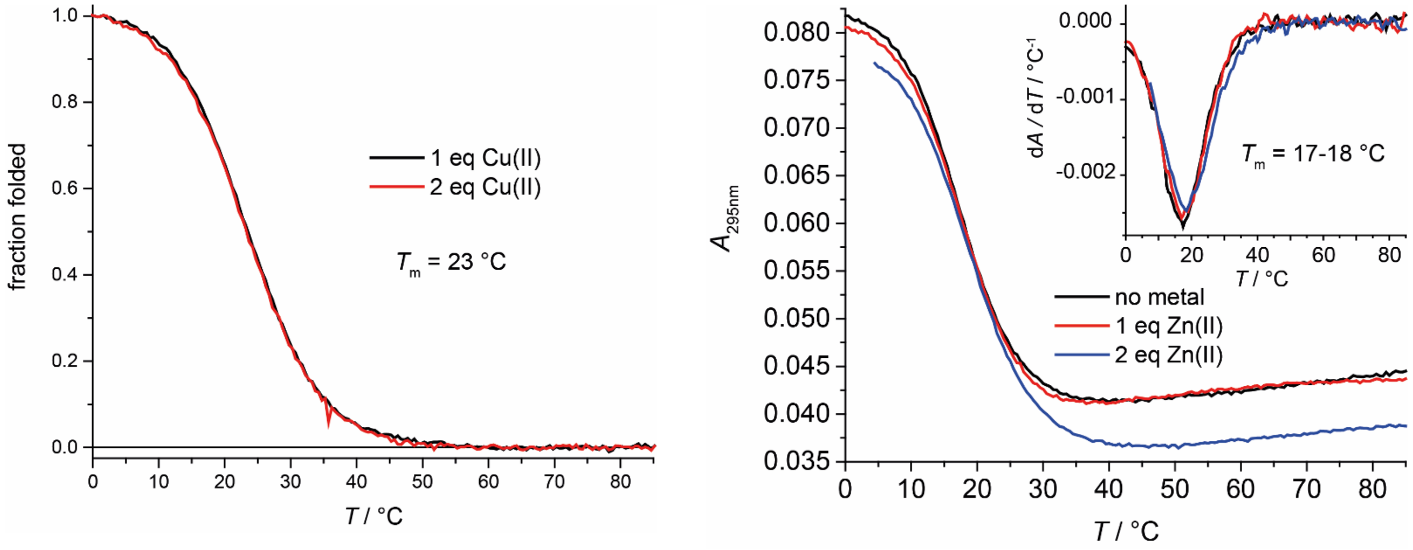

Fig. S21: Thermal denaturation profiles of G-quadruplex htel$\mathbf{L^B}_4\mathbf{L^I}_2$ in absence or presence of 1 or 2 equiv. $CuSO_4$ or ZnI_2. A thermal stabilization of ΔT_m = 6 °C could be observed after addition of Cu(II) whereas no stabilization could be observed after addition of Zn(II). Due to a low melting temperature, in absence of a transition metal ion, no fraction folded curve could be generated.

htel$\mathbf{L^B}_3\mathbf{L^I}_3$

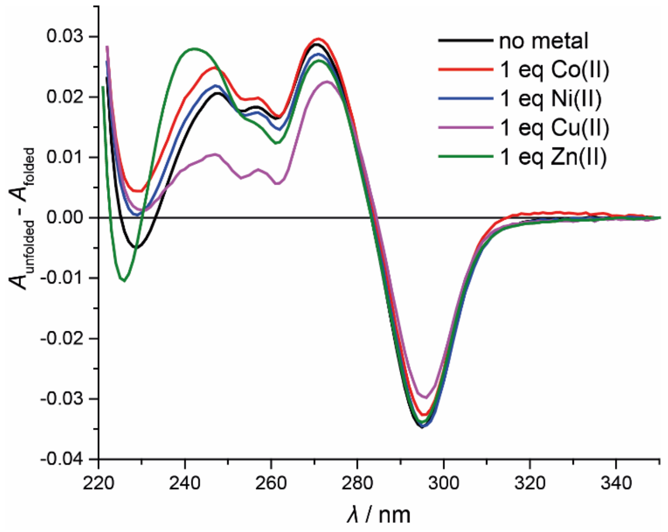

Fig. S22: Thermal difference spectra of G-quadruplex htel$\mathbf{L^B}_3\mathbf{L^I}_3$ in absence or presence of 1 equiv. $Co(NO_3)_2$, $NiSO_4$, $CuSO_4$ or ZnI_2.

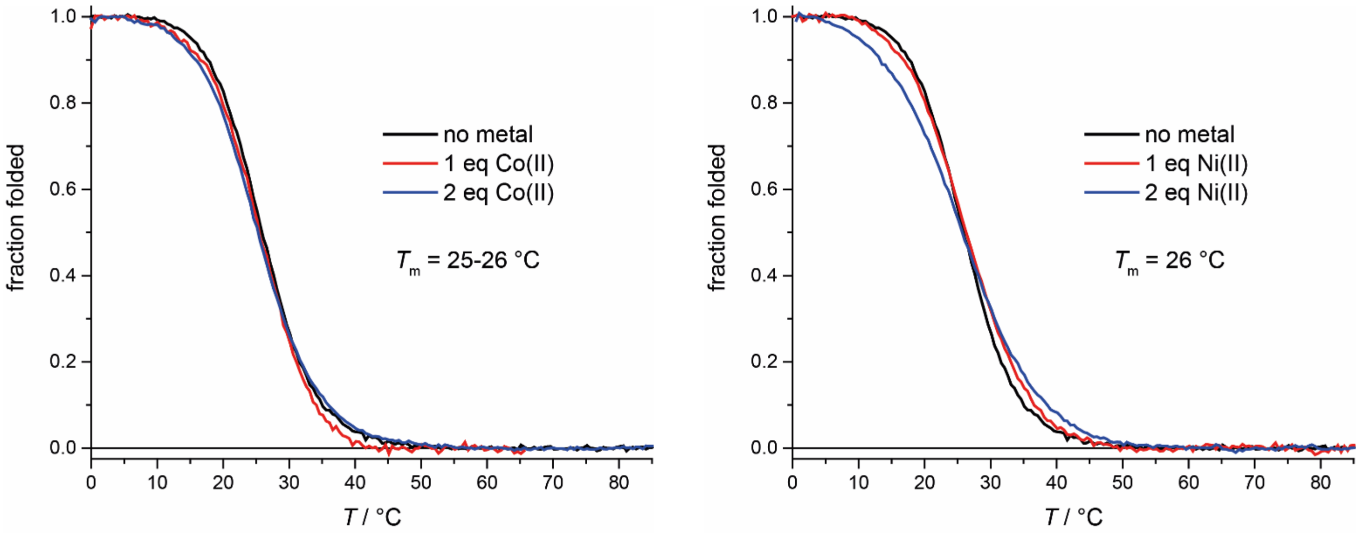

Fig. S23: Thermal denaturation profiles of G-quadruplex htel$\mathbf{L}^{B}_{3}\mathbf{L}^{I}_{3}$ in absence or presence of 1 or 2 equiv. $Co(NO_3)_2$ or $NiSO_4$. No thermal stabilization could be observed after addition of the transition metal ions.

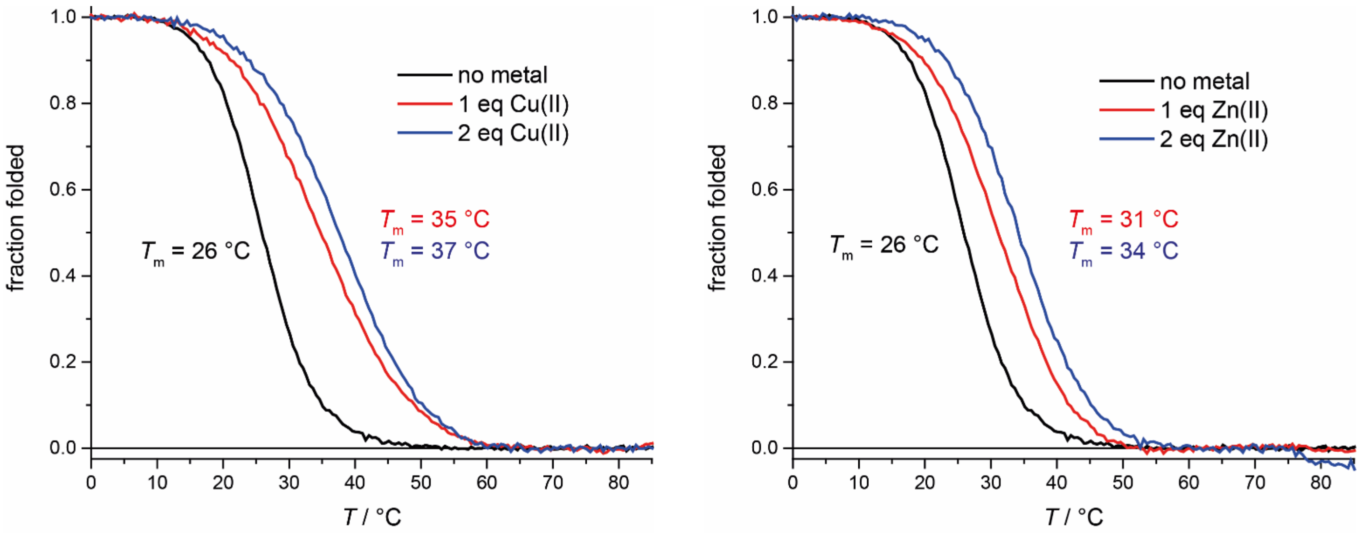

Fig. S24: Thermal denaturation profiles of G-quadruplex htel$\mathbf{L}^{B}_{3}\mathbf{L}^{I}_{3}$ in absence or presence of 1 or 2 equiv. $CuSO_4$ or ZnI_2. Thermal stabilizations of ΔT_m = 9 °C after addition of Cu(II) and of ΔT_m = 5 °C after addition of Zn(II) could be observed.

6 CD Spectroscopy

6.1 Sample Preparation

For CD measurements, the samples were prepared in the same way as for the UV-based thermal denaturation studies (chapter 5.1).

6.2 Spectrometer and Methods

CD spectra were recorded on an *Applied Photophysics Chirascan qCD spectropolarimeter* (350 – 205 nm, 0.5 s time-per-point, step size 1 nm, bandwidth 0.5 nm, 3 repeats) at 7 °C. Temperature was controlled using a *Quantum Northwest* temperature control attached to a sample probe. The background was measured in the same cuvette as the sample. To avoid condensation of water onto the cuvette surface or cell window, a constant nitrogen gas flow was maintained.
All spectra were averaged, smoothed (Savitzky-Golay, window size 5), background corrected (cuvette, buffer and electrolyte; the background spectrum was smoothed before (Savitzky-Golay, window size 10)) and zeroed to the signal at 350 nm.

6.3 CD Spectra

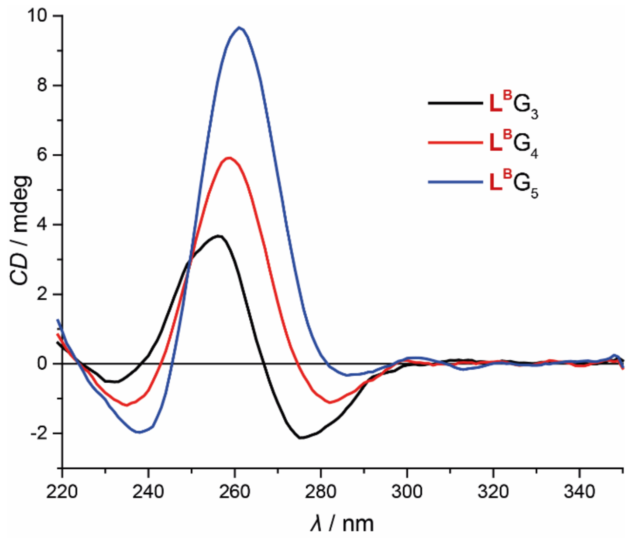

Fig. S25: CD spectra of $\mathbf{L^B}G_n$ (n = 3-5). For $\mathbf{L^B}G_3$, no formation of a G-quadruplex structure is observed. For $\mathbf{L^B}G_4$ and $\mathbf{L^B}G_5$, the CD spectra clearly indicate the formation of parallel G-quadruplex structures $(\mathbf{L^B}G_4)_4$ and $(\mathbf{L^B}G_5)_4$.

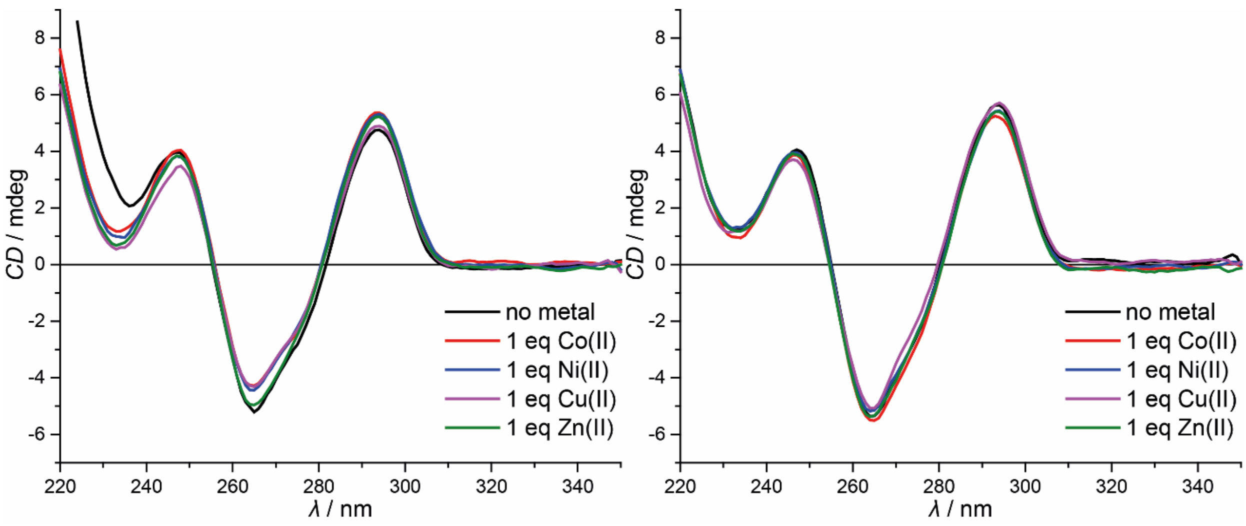

Fig. S26: CD spectra of folded G-quadruplex htel$\mathbf{L}^{\mathbf{B}}{}_{4}$ (left) and htel$\mathbf{L}^{\mathbf{B}}{}_{3}\mathbf{L}^{\mathbf{I}}$ (right) in absence or presence of 1 equiv. $Co(NO_3)_2$, $NiSO_4$, $CuSO_4$ or ZnI_2.

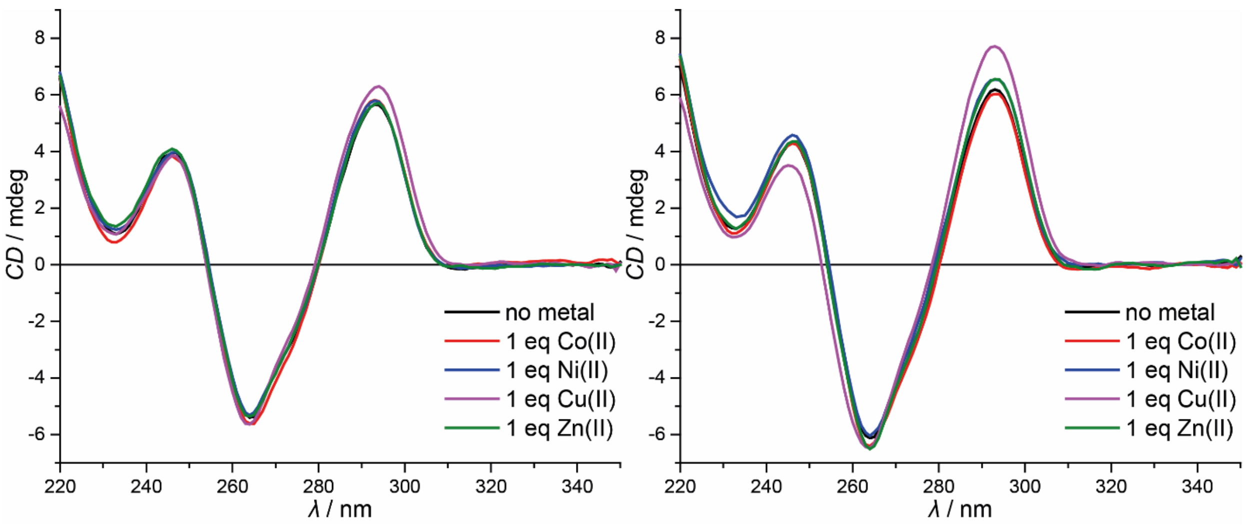

Fig. S27: CD spectra of folded G-quadruplex htel$\mathbf{L}^{\mathbf{B}}{}_{2}\mathbf{L}^{\mathbf{I}}{}_{2}$ (left) and htel$\mathbf{L}^{\mathbf{B}}\mathbf{L}^{\mathbf{I}}{}_{3}$ (right) in absence or presence of 1 equiv. $Co(NO_3)_2$, $NiSO_4$, $CuSO_4$ or ZnI_2.

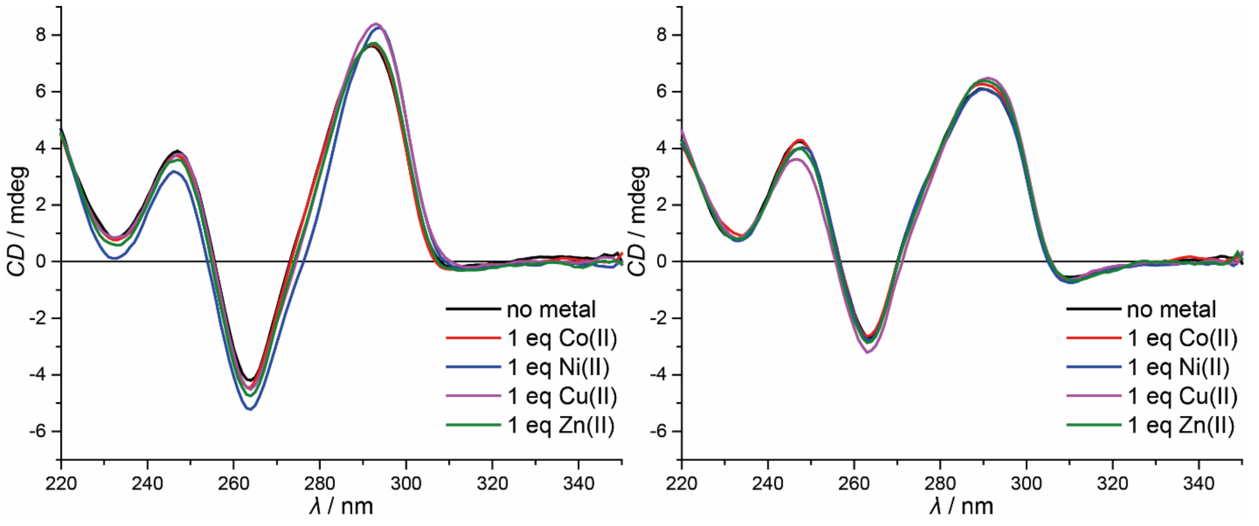

Fig. S28: CD spectra of folded G-quadruplex htel$\mathbf{L}^{\mathbf{B}}{}_{2}\mathbf{L}^{\mathbf{I}}{}_{4}$ (left) and htel$\mathbf{L}^{\mathbf{B}}{}_{4}\mathbf{L}^{\mathbf{I}}{}_{2}$ (right) in absence or presence of 1 equiv. $Co(NO_3)_2$, $NiSO_4$, $CuSO_4$ or ZnI_2.

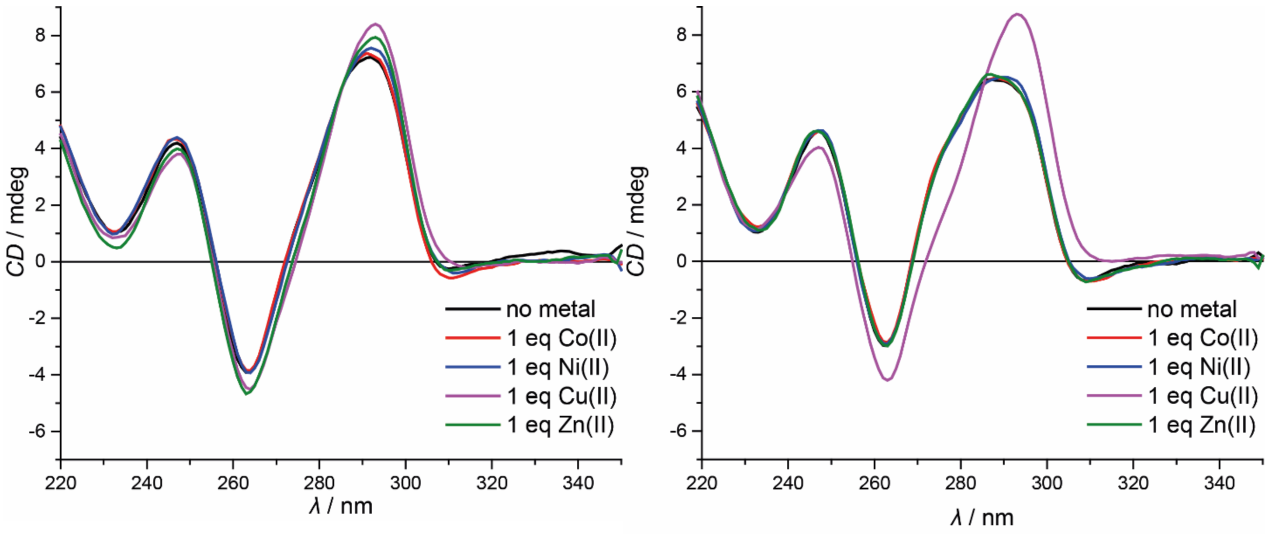

Fig. S29: CD spectra of folded G-quadruplex htel$\mathbf{L}^{\mathbf{B}}{}_{3}\mathbf{L}^{\mathbf{I}}{}_{3}$ (left) and htel$\mathbf{L}^{\mathbf{I}}{}_{4}$B(right) in absence or presence of 1 equiv. $Co(NO_3)_2$, $NiSO_4$, $CuSO_4$ or ZnI_2.

7 Native ESI Mass Spectrometry

7.1 Sample Preparation

For native ESI MS studies, G-quadruplex samples containing 25 µM DNA, 1 mM KCl, 100 mM TMAA buffer pH 7.0 and 25 µM $NiSO_4$ or $CuSO_4$ were prepared. Samples were heated to 85 °C for 10 min, slowly cooled to 4 °C with a cooling rate of 0.5 °C/min and then left at this temperature for several hours (typically overnight). Prior to measurement, samples were diluted with acetonitrile (1:1 v/v).

7.2 Spectrometer and Methods

Native ESI mass spectrometry was performed on a *Bruker ESI-timsTOF* mass spectrometer (negative mode, capillary voltage: 4500 V, end plate offset voltage: 500 V, nebulizer gas pressure: 0.4/0.6 bar, dry gas flow rate: 6/9 L/min, dry temperature: 303 K). For calibration of the TOF device, *Agilent ESI-Low Concentration Tuning Mix* was used.

7.3 Native ESI Mass Spectra

In native ESI MS, the secondary structure is kept intact. To differentiate between folded and unfolded G-quadruplexes in the gas phase, the following phenomenon is most instructive: ESI mass spectrometry from potassium-containing solutions always gives rise to series of unspecific adducts with potassium cations. For fully denatured species, a statistical distribution of adducts starting with zero potassium ions would be observed and for a folded species, a distribution is observed starting with n-1 explicitly bound cations where n is the number of G-tetrads.

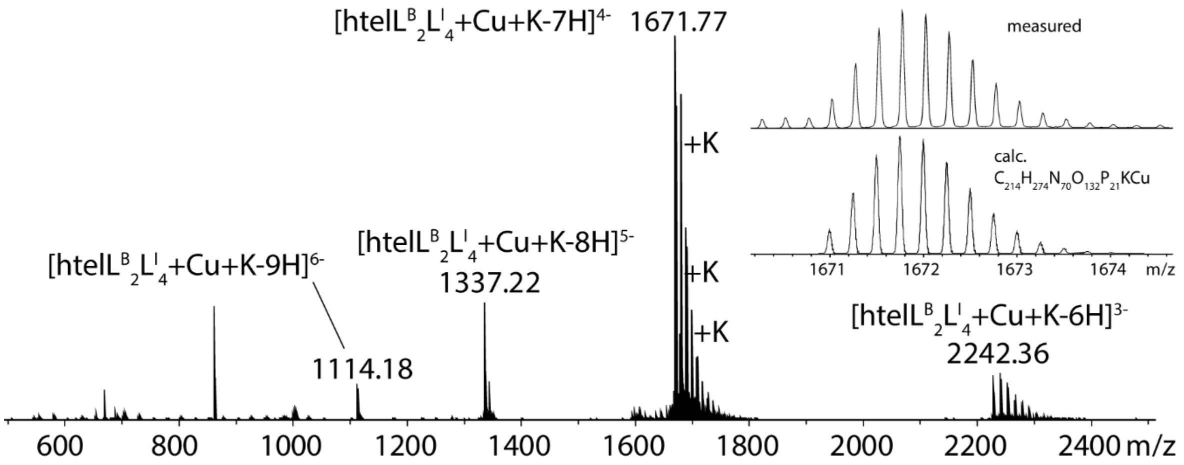

Fig. S30: Native ESI-MS spectrum of htel$\mathbf{L^B}_2\mathbf{L^I}_4$ with 1 equiv. Cu(II). A distribution of potassium adducts starting with n-1 explicitly bound cations is observed, whereas a species without potassium is not observed. This suggests a folded G-quadruplex species in the gas phase.

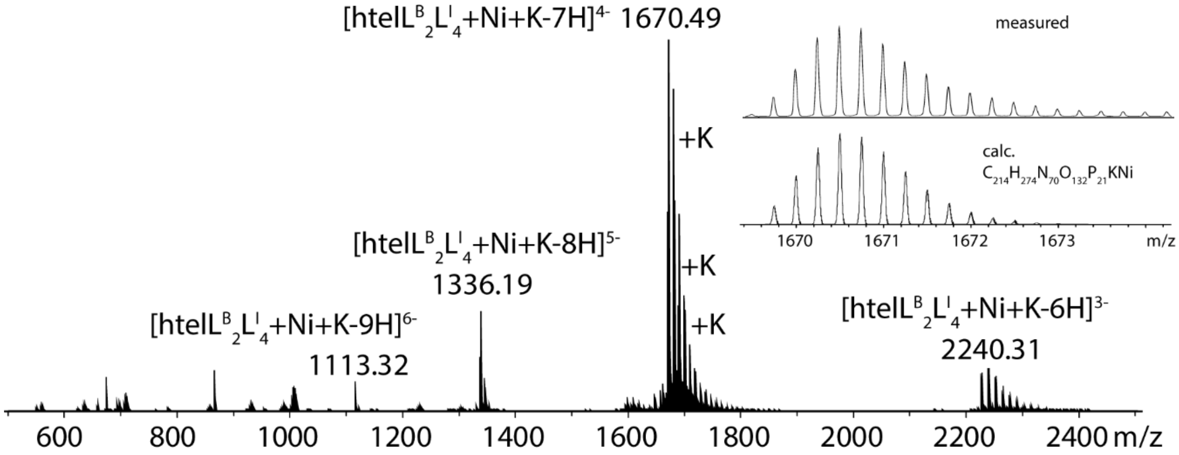

Fig. S31: Native ESI-MS spectrum of htel$\mathbf{L^B}_2\mathbf{L^I}_4$ with 1 equiv. Ni(II). A distribution of potassium adducts starting with n-1 explicitly bound cations is observed, whereas a species without potassium is not observed. This suggests a folded G-quadruplex species in the gas phase.

8 Mixtures of Ligands in Tetramolecular G-Quadruplexes

Mixing ligands in tetramolecular G-quadruplexes leads to statistical mixtures or narcissistic self-sorting, which makes it challenging to design distinct heteroleptic coordination environments.

Note that in these experiments, the (*R*) enantiomer of $\mathbf{L}^{\mathrm{I}}$ was used.

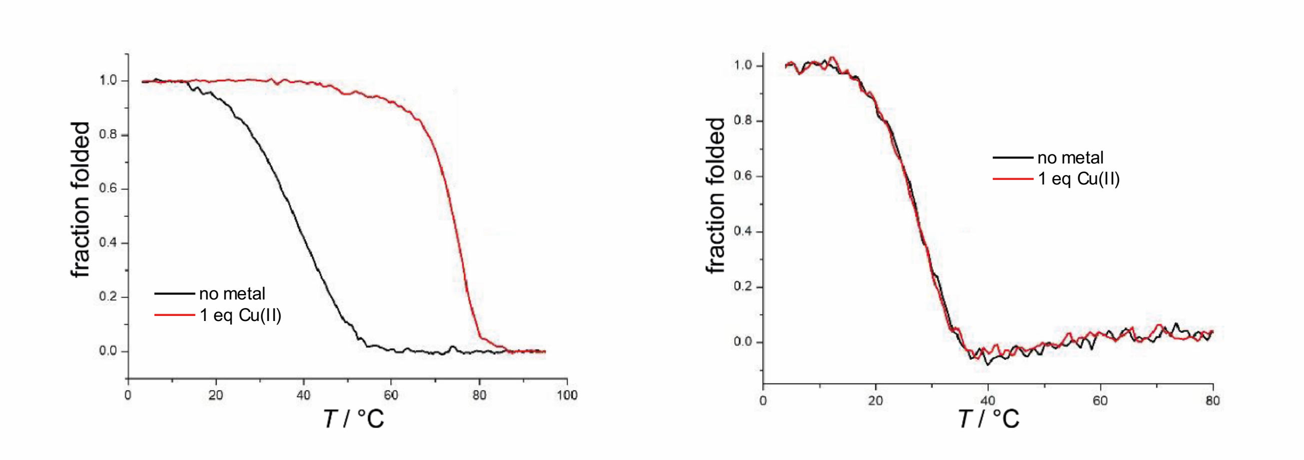

Fig. S32: Thermal denaturation profiles of $(\mathbf{L}^{\mathrm{I}}G_4)_4$ (left) and $(\mathbf{L}^{\mathrm{B}}G_4)_4$ (right) in absence or presence of 1 equiv. $CuSO_4$.

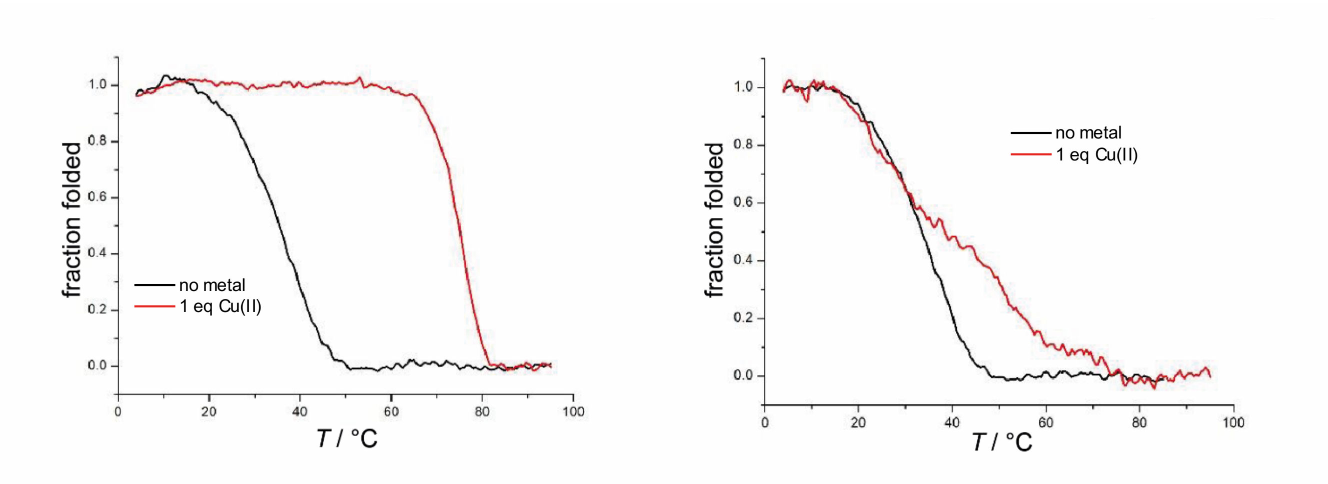

Fig. S33: Thermal denaturation profiles of mixtures of G-quadruplexes composed of oligonucleotide $\mathbf{L}^{\mathrm{I}}G_4$ and $\mathbf{L}^{\mathrm{B}}G_4$ in absence or presence of 1 equiv. $CuSO_4$. With an oligonucleotide ratio of $\mathbf{L}^{\mathrm{I}}G_4$:$\mathbf{L}^{\mathrm{B}}G_4$ 3:1 (left), narcissistic self-sorting occurs after addition of Cu^{II}. With an oligonucleotide ratio of $\mathbf{L}^{\mathrm{I}}G_4$:$\mathbf{L}^{\mathrm{B}}G_4$ 1:3 (right), a statistical mixture is obtained after addition of Cu^{II}.

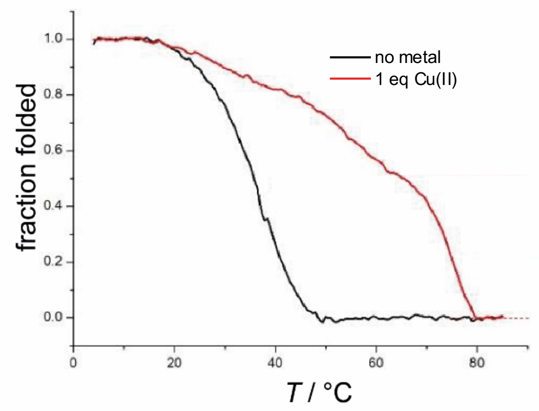

Fig. S34: Thermal denaturation profiles of mixtures of G-quadruplexes composed of oligonucleotide $\mathbf{L}^{I}G_4$ and $\mathbf{L}^{B}G_4$ in absence or presence of 1 equiv. $CuSO_4$. With an oligonucleotide ratio of $\mathbf{L}^{I}G_4$:$\mathbf{L}^{B}G_4$ 2:2, a statistical mixture is obtained after addition of Cu^{II}.

9 References

Dolomanov, O. V., Bourhis, L. J., Gildea, R. J., Howard, J. A. K., and Puschmann, H. (2009). OLEX2: a complete structure solution, refinement and analysis program. *J. Appl. Crystallogr.* 42, 339–341. doi:10.1107/s0021889808042726

Engelhard, D. M., Nowack, J., and Clever, G. H. (2017). Copper-Induced Topology Switching and Thrombin Inhibition with Telomeric DNA G-Quadruplexes. *Angew. Chem. Int. Ed.* 56, 11640–11644. doi:10.1002/anie.201705724

Engelhard, D. M., Pievo, R., and Clever, G. H. (2013). Reversible Stabilization of Transition-Metal-Binding DNA G-Quadruplexes. *Angew. Chem. Int. Ed.* 52, 12843–12847. doi:10.1002/anie.201307594

Farrugia, L. J. (1997). ORTEP-3 for Windows - a version of ORTEP-III with a Graphical User Interface (GUI). *J. Appl. Crystallogr.* 30, 565–565. doi:10.1107/s0021889897003117

Mergny, J., and Lacroix, L. (2009). UV Melting of G-Quadruplexes. *Curr. Protoc. Nucleic Acid Chem.* Chapter 17, 17.1.1-17.1.15. doi:10.1002/0471142700.nc1701s37

Mergny, J.-L., Li, J., Lacroix, L., Amrane, S., and Chaires, J. B. (2005). Thermal difference spectra: a specific signature for nucleic acid structures. *Nucleic Acids Res.* 33, e138–e138. doi:10.1093/nar/gni134

Punt, P. M., and Clever, G. H. (2019). Tailored Transition-Metal Coordination Environments in Imidazole-Modified DNA G-Quadruplexes. *Chem. Eur. J.* 25, 13987–13993. doi:10.1002/chem.201903445

Sheldrick, G. M. (2008). A short history of SHELX. *Acta Crystallogr. Sect. A Found. Crystallogr.* 64, 112–122. doi:10.1107/s0108767307043930

Sheldrick, G. M. (2015). SHELXT – Integrated space-group and crystal-structure determination. *Acta Crystallogr. Sect. A Found. Crystallogr.* 71, 3–8. doi:10.1107/s2053273314026370

Zhai, Q., Deng, M., Xu, L., Zhang, X., and Zhou, X. (2012). Promoting the formation of tetramolecular G-quadruplexes under freezing condition. *Bioorg. Med. Chem. Lett.* 22, 1142–1145. doi:10.1016/j.bmcl.2011.11.099
